# High support housing for people with serious mental illness in Canada: a scoping review

**DOI:** 10.3389/fpsyt.2025.1612516

**Published:** 2025-06-24

**Authors:** Alexandra Sosnowski, Mariana Ravazolli Martins, Eric A. Latimer, Frank Sirotich, Julia Woodhall-Melnik, Nick Kerman, Vicky Stergiopoulos

**Affiliations:** ^1^ Centre for Addiction and Mental Health, Toronto, ON, Canada; ^2^ Douglas Mental Health University Institute, Montréal, QC, Canada; ^3^ Department of Psychiatry, McGill University, Montréal, QC, Canada; ^4^ Canadian Mental Health Association Toronto Branch, Toronto, ON, Canada; ^5^ Factor-Inwentash Faculty of Social Work, University of Toronto, Toronto, ON, Canada; ^6^ Department of Social Science, University of New Brunswick, Saint John, NB, Canada; ^7^ Department of Psychiatry, University of Toronto, Toronto, ON, Canada

**Keywords:** supportive housing, high support housing, housing first, mental illness, homelessness, scoping review, Canada

## Abstract

**Introduction:**

Mental health supportive housing aims to provide accommodation and support services to people with serious mental illness (SMI). Various supportive housing models have emerged in Canada post-deinstitutionalization, with heterogeneous and limited available information on congregate-based high support housing models (HSH) that provide 24/7 onsite supports. A registered scoping review was undertaken to identify the models and outcomes of HSH for people with SMI in Canada, including those exiting homelessness.

**Methods:**

Four academic databases (Medline, Embase, PsycINFO, CINAHL Plus) were searched, in addition to backward and forward citation searching and strategies for identifying grey literature. HSH configurations and outcomes (e.g., housing stability and preferences; physical and behavioral health; community integration, social support, and quality of life; costs) were investigated within the Canadian context.

**Results:**

A total of 27,180 academic articles and 164 grey literature reports were initially screened. Following full-text review of 337 academic and 109 grey literature articles, and citation searching, a total of 58 academic articles and 31 grey literature reports were included in the review. The characteristics, objectives, and outcomes of HSH models were mixed. Three nonexclusive types of HSH were identified: [1] custodial HSH, [2] recovery-oriented HSH, and [3] alternatives to hospital programs and other institutions. Most studies were uncontrolled, though there was preliminary evidence to support improvements in HSH residents' health and functioning; gains in social support, quality of life, and community integration; reductions in housing and service costs, particularly when compared to the cost of inpatient hospitalization; and satisfaction with living arrangements, supports, and relationships. However, the findings were not unequivocal, and the diversity of methodologies and HSH models limited further comparisons of outcomes between the different types of HSH.

**Conclusion:**

People with SMI experiencing complex and diverse needs can benefit from the services and supports provided by HSH. However, research on HSH is less developed compared to other, well-studied housing interventions. Further research on congregate housing settings, including their long-term outcomes and recovery-oriented practices, is warranted.

## 1 Introduction

Housing and community-based services have assumed central roles in supporting people with serious mental illness (SMI) following deinstitutionalization, with different housing and service bundles emerging organically over time (1–4). Mental health supportive housing programs (hereinafter referred to as "supportive housing") generally aim to provide accommodation and flexible supports to address acute and chronic health conditions, improve practical living skills and social functioning, and promote recovery and independence among people with SMI (3–7). These programs are often targeted to people being discharged from hospital, exiting homelessness, or leaving jail or other institutions.

In-depth international study of supportive housing models has been hindered by confusion about program labels, and variations in physical structures and provided supports, presenting obstacles to study and synthesis attempts (3, 4, 7, 8). Further, the quality of housing and services, including their recovery orientation, has varied over time within and across jurisdictions (1, 3, 8–12). Nonetheless, despite considerable heterogeneity, supportive housing has generally been found to be effective across a range of outcomes, including housing stability and appropriate use of health services, although few studies were controlled (2, 4, 6, 10–18). Among the range of supportive housing models, *Housing First* (HF) has been the most rigorously examined (3, 19–22). HF typically offers immediate access to scattered-site housing through rent supplements and off-site supports to individuals with histories of homelessness and mental illness (23). Less is known about congregate (i.e., single-site) supportive housing models, particularly high support housing (HSH).

There is no consensus on the components of HSH, but these programs generally offer 24-hour staffing and structured supports within a congregate setting for people with SMI and severe disabilities; meals, personal support, and support with medication may also be provided. A range of HSH programs have been described in the international literature, ranging from short-term programs that aim to stabilize patients in crisis or post-hospitalization, to permanent housing that offers long-term, stable accommodation aimed at promoting independent living and participation in meaningful activities (2, 3, 17, 24–26). Not surprisingly, terms and definitions used to describe HSH are variable and inconsistent across jurisdictions, as are program models and philosophies, with Italy, Australia, and the United Kingdom among the countries with well-described housing models for adults with SMI (13–17, 27, 28). Internationally, reported outcomes associated with HSH have been mixed; some studies contend that HSH may be helpful in promoting safety, stability, and social interaction among residents, whereas others view HSH as restrictive, undermining resident recovery, choice, and autonomy (11, 29–31). Despite evidence of self-determination concerns, HSH is nonetheless an important type of supportive housing for people with SMI who cannot live independently in the community.

Within Canada, HSH configurations include residential care homes, board-and-care homes, and group and custodial homes, among others. Group homes tend to be smaller structured settings, whereas residential care homes vary in size and program philosophy, with some adopting a custodial orientation, and others employing trained mental health staff to support residents' psychosocial rehabilitation. In contrast, board-and-care homes are primarily for-profit operations, and typically employ unregulated staff with limited training related to mental health. Notably, as community mental health services, including HSH, are overseen provincially, rather than federally, there is great geographical variation of HSH funding models, programs structures, and resources across the country, with lack of standards to guide delivery in most settings.

As of 2011, an estimated 520,700 people with mental illness in Canada did not have access to adequate housing, and over 119,800 were experiencing homelessness (32). A study of adults experiencing chronic homelessness in Toronto estimated that approximately 9% needed 24-hour support in a residential care facility (33). Furthermore, approximately 60% of long-stay psychiatric inpatients who no longer require hospitalization, but are awaiting discharge to more appropriate settings, could be transitioned to HSH, if it were available (34–36). A more recent analysis found that as many as 20% of inpatients in a large psychiatric hospital in Toronto are awaiting HSH at any given time, delaying discharge and prolonging institutionalization (37). In most settings in Canada, however, HSH programs are considered in short supply, with little evidence to guide planning and program development.

Given the dearth of knowledge on HSH for people with SMI in Canada, a scoping review was undertaken to identify available models, program philosophies, and outcomes of HSH in this country. A scoping review was selected based on a preliminary search of the literature that revealed limited and heterogenous research on HSH in Canada. The scoping review had two research questions: [1] How is HSH conceptualized and described in the Canadian literature?; and [2] What are the reported resident characteristics, outcomes, experiences, and associated costs of HSH settings in Canada?

## 2 Methods

The design of this scoping review was based on the framework developed by Arksey and O'Malley (38), and adhered to reporting guidelines of the Preferred Reporting Items for Systematic reviews and Meta-Analyses extension for scoping reviews (PRISMA-ScR; see **Supplementary Table 1**) (39). A protocol for the review was developed prior to initiation, and was registered with Open Science Framework (OSF) at the point of evidence selection (https://doi.org/10.17605/OSF.IO/UHRD7).

### 2.1 Inclusion criteria

The inclusion criteria are described following the Population, Concept, Context mnemonic (40). The *population* of interest was: adults (≥18 years) with SMI (i.e., mental illnesses, including depressive disorders, bipolar disorders, and schizophrenia, that significantly impair daily functioning and limit life activities) (41), including those exiting homelessness. For studies with mixed or general samples of people with mental illness, ≥ 50% of participants were required to have an SMI for inclusion in the review. The intervention, a core *concept*, was defined as congregate housing programs that had a minimum duration of 3-months, with the presence of 24-hour, onsite supports. For studies that examined multiple interventions or included findings that were not differentiated by housing type, ≥50% of programs were required to be HSH, as described above. A range of outcomes were identified in the examined literature: housing stability; housing satisfaction and preferences; physical and behavioral health; community integration, social support, and quality of life; costs; offending and recidivism; health service use; and housing supply and demand needs. The *context* was HSH in Canada, with an examination of different geographical locations, service configurations, and subpopulations of adults with SMI, such as forensic mental health and long-stay hospital patients. If program or sample details were insufficient for determining eligibility, the research team contacted the corresponding authors for further information or clarification on potentially relevant work.

Both published academic and grey literature were eligible for this scoping review. Academic articles were inclusive of empirical studies, commentaries, program evaluations and descriptions, as well as conceptual or overview articles published in peer-reviewed journals. Grey literature was limited to technical reports; policy, advocacy, and assessment documents; and organizational documents describing programs and/or frameworks. Articles were required to be published between January 1, 1990 and July 19, 2023 (including advanced online academic articles), and written in either English or French.

Exclusion criteria were: conference abstracts, dissertations and theses, news media articles, study protocols, and literature pertaining to long-term care or housing for individuals with intellectual and developmental disabilities, as well as those related to individuals with substance use disorders without co-occurring SMI. Articles with duplicative information as other sources in the review were excluded (the document with the most detailed information was prioritized for inclusion).

### 2.2 Search strategy

Eligible academic articles were systematically sourced from four databases: [1] Medline (Ovid), [2] APA PsycINFO (Ovid), [3] Embase (Ovid), and [4] CINAHL Plus (EBSCO). A comprehensive search was first developed in Medline (Ovid; **Table 1**) and later adapted to other databases. Database selection and the development of the search strategy was performed in consultation with an experienced librarian at the Centre for Addiction and Mental Health, and strategies were further reviewed and revised by senior team members (VS, NK). Additional articles were identified through backward and forward citation searching. See **Supplementary Tables 2**-**4** for detailed search strategies in other databases.

**Table 1:** Ovid MEDLINE Search Strategy.

Ovid MEDLINE: Epub Ahead of Print, In-Process & Other Non-Indexed Citations, Ovid MEDLINE^®^ Daily and Ovid MEDLINE^®^ <1946-Present>		
1	Mental Disorders/	177801
2	Mentally Ill Persons/	643
3	((complex or severe* or serious* or persist* or chronic* or significant*) adj (mental* or psychiatr* or psycholog*)).ti,ab,kf.	31391
4	Ill-Housed Persons/	9808
5	(ill-housed or unhoused or street people or street dwelling or shelter* or homeless* or forensic*).ti,ab,kf.	81758
6	(alternat* level* of care or alternat* care).ti,ab,kf.	913
7	or/1-6	281211
8	Tertiary Healthcare/	1829
9	Residential Treatment/	3325
10	Housing/	20153
11	residential facilities/or assisted living facilities/or group homes/or halfway houses/	9235
12	((support* or permanent or resident* or assisted or custodial or group or communit*) adj2 (hous* or home* or accommodation* or service* or unit* or facilit* or living*)).ti,ab,kf.	96708
13	(hous* adj2 (program* or intervention* or service* or model*)).ti,ab,kf.	4712
14	or/8-13	128664
15	7 and 14	10339

A separate search strategy was developed for grey literature. Informed by Godin and colleagues (42), the search strategy included: [1] targeted searches and browsing of websites (e.g., Homeless Hub, Canadian Mental Health Association, Wellesley Institute), [2] customized Google searches, and [3] government document and grey literature databases (e.g., Canadian Research Index, Publications Canada, Health Canada). Backward and forward citation searching was again used to identify additional literature.

### 2.3 Screening

Following the search, all identified citations were collated and imported into Covidence for screening. Screening at the title- and abstract-levels (accompanying summaries for grey literature) was performed independently by two research team members (AS, MRM). At the full-text phase, two members (AS, MRM) screened each article against eligibility criteria. The senior responsible author also assessed articles selected at this phase in full. Throughout the screening process, the two reviewers met regularly with the senior authors (NK, VS) to discuss progress and resolve conflicts, leveraging the expertise of other team members to reach consensus when

### 2.4 Data extraction and data synthesis

A data extraction form was developed to document the following components of included articles: [1] title and authors; [2] publication year; [3] location of the research/housing program; [4] study objectives and goals; [5] details on the housing and support model, including available services, target population, and program philosophy/values; [6] sample characteristics; and [7] outcomes of interest. Two research team members piloted the extraction template on 3–5 articles to familiarize themselves with the template, and to ensure consistent charting. The same members continued to extract information independently on assigned articles, which were subsequently reviewed by others on the research team.

As the aim of the scoping review was to identify the literature on HSH in Canada, critical appraisal of the included articles was not performed. Academic and grey literature were analyzed together, using a narrative approach. This entailed a descriptive summary of included studies (**Supplementary Tables 7** and **8**), considerations of study rigor, and exploration of relationships between studies to identify shared elements and group identified housing models into categories, leveraging program descriptions and underlying philosophies. Priority in reporting outcomes was given to academic articles describing experimental and quasi-experimental studies.

## 3 Results

**Figure 1:**
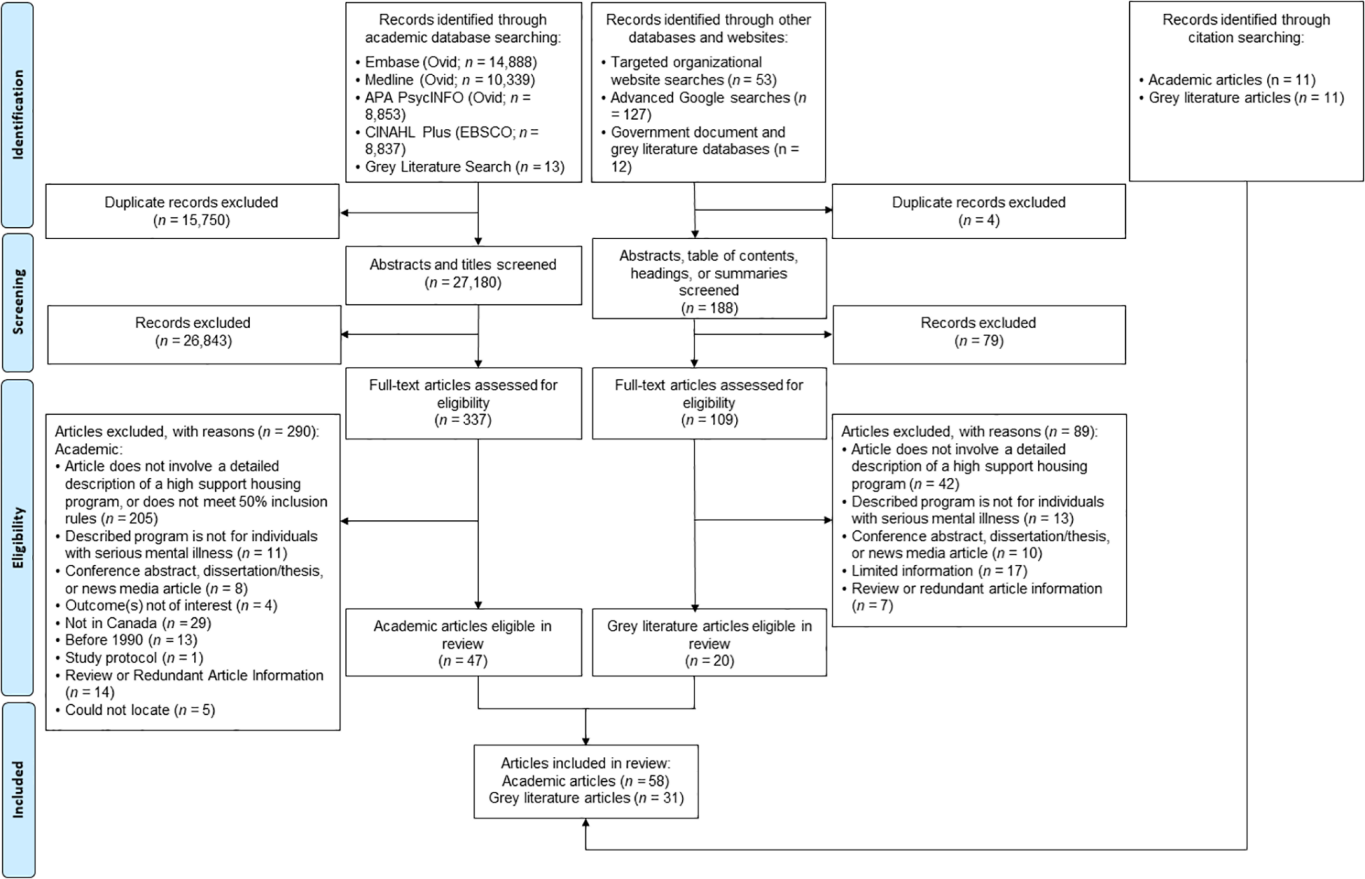
PRISMA Flowchart.

The record identification process and outcomes are shown in **Figure 1**. Academic database searches yielded 27,180 non-duplicative articles, of which 337 underwent full-text review. A total of 58 academic articles were included in the review (including 11 articles identified through citation searching; **Figure 1**). Articles included qualitative studies (n=17); cross-sectional studies (n=12); overviews, commentaries, and conceptual articles (n=7); randomized controlled trials (RCTs; n=7); and case studies and program evaluations/descriptions (n=6). Of note, the seven articles describing an RCT design were from the same parent study. The articles focused on four provinces: Ontario (n=22), Quebec (n=18), British Columbia (n=9), and New Brunswick (n=1). One article examined two provinces (Quebec and British Columbia), and seven others were not specific to any province or territories. See **Supplementary Table 5** for an overview on the individual academic articles.

Non-academic and website searches yielded 192 potentially relevant records, with 164 added into Covidence for full-text review. A total of 31 grey literature articles were included in the scoping review. The majority of the grey literature examined HSH in Ontario (n=18), followed by British Columbia (n=4), Alberta (n=4), Nova Scotia (n=1), and Quebec (n=1); three documents did not specify a single or set of provinces or territories. See **Supplementary Table 6** for an overview on the individual grey literature articles.

### 3.1 Supportive model types, philosophies, and objectives

The characteristics and services delivery models, philosophies, and objectives of HSH varied considerably across the included literature. These were synthesized, based on aims and program philosophy, to identify three core types of HSH: [1] *custodial HSH*; [2] *recovery-oriented HSH*; and [3] *alternatives to hospital programs and other institutions*. Notably, these three categories were not mutually exclusive. There was insufficient information on program models in some work; these articles were retained in the review, but not categorized (43–45).

#### 3.1.1 Custodial HSH

Thirty-eight articles (academic literature: n=30; grey literature: n=8) discussed or examined *custodial HSH* models. This encompassed board-and-care homes (46–59), foster homes for adults with SMI in Quebec (60–71), as well as hostels and select group homes (58, 71–77). Generally, these housing models were privately operated and for-profit, aiming to maintain resident stability by delivering around-the-clock accommodations, meals, and other support services (e.g., housekeeping, laundry services) (32, 46, 48, 49, 52–55, 57, 78).

Among the earliest HSH models, custodial housing was historically framed as a home-like alternative to institutional psychiatric care, but has long been criticized for perpetuating features of institutionalization (47, 49–51, 62, 78). Further, such HSH models were described as lacking formal rehabilitation programming and neglecting resident choice, autonomy, and personal growth (48–55, 59, 78). Non-professional staff and supervision was typical of custodial housing (49, 58, 78); for example, no training was required to become a caregiver in foster homes for adults with SMI in Quebec, wherein multidisciplinary teams acted as linkages between the home and formal health services (64, 65, 67–70). In qualitative studies, these caregivers self-identified as "both the parents and rehabilitation agents" of their foster homes (64), as well as "dedicated helpers." (69)

A few such traditionally custodial programs were described as beginning to implement practices to promote recovery and community integration, to better address resident needs and preferences, highlighting how policy mandates were being used to facilitate a transition from custodial into more recovery-oriented HSH (47, 49–51). This included a shift away from *Homes for Special Care* to *Community Homes for Opportunity* in Ontario, for example, to better align program philosophy with current best practices; the newer iterations are reportedly more recovery-oriented, with enhanced professional supports and person-centered approaches (47, 50, 51).

#### 3.1.2 Recovery-oriented HSH

Thirty-four articles (academic literature: n=19; grey literature: n=15) examined *recovery-oriented HSH.* These programs were typically operated by non-profit agencies and had no restriction on residents' length of stay (i.e., offered permanent housing). In addition to stand-alone programs, this type of HSH also included groups such as *Community Homes for Opportunity* and a single-site HF program (47, 50, 51, 79–85). Overall, these programs were more recovery-oriented, aiming to promote resident choice, autonomy, goal orientation and achievement. Various supports and leisure activities were typically offered within the housing programs or in their surrounding communities (e.g., employment programs/opportunities, resource and financial management) (32, 47, 50, 51, 84–90). Further, a harm reduction orientation was referenced, most notably among programs with a HF philosophy, wherein tenancy is not contingent on adherence to treatment or abstinence from substances (83–86, 89–92).

Unlike custodial housing, program supports and services were typically delivered by qualified clinical and non-clinical staff, such as case managers and peer support workers. Staff assisted residents with a range of tasks, including personal and life skills supports, social and health services system navigation, managing medication, and care coordination in partnership with external community and health providers (47, 49–51, 82, 86–88, 91, 92). In some programs, staff also facilitated house meetings, during which conflicts were mediated and expectations for communal living discussed (87, 88, 93–95). Recognizing that individual needs fluctuate over time, a few housing programs described varying and graduating levels of support and supervision, with residents moving to higher or lower levels of support as needed (87, 88).

#### 3.1.3 Alternatives to hospital and other institutions

Nineteen articles (academic literature: n=10; grey literature: n=9) examined or described *alternatives to hospital and other institutions*. This category included: [1] step-down programs that operated at arms-length from a discharging hospital (37, 96–98); [2] tertiary psychiatric residential facilities (18, 24, 99–102); and [3] transitional housing programs serving forensic mental health patients (34, 103–107). Generally, these programs aimed to prepare individuals for eventual move to more independent, longer-term accommodations within the community. Person-centered, recovery-oriented approaches were frequently described in relation to housing programs in this category (97–99, 102–104).

Programs typically served a specific population of people with SMI, including individuals with support needs exceeding the expertise and resources of other settings (18, 24, 34, 99–102); patients with prolonged psychiatric hospitalizations (34, 37, 96, 98); and forensic mental health patients discharged from hospital (34, 103–107). In these settings, specialized services and supports were delivered by multi-disciplinary teams with low resident to staff ratios. Available supports were typically wide-ranging, including medication management, peer supports, nursing and psychiatric services, substance use and behavioral supports, as well as recreational, vocational, and life skills trainings (97–101, 103, 104, 106).

### 3.2 Resident characteristics

The target population of HSH programs, as per program descriptions, was persons with SMI, including those with "complex needs," as well as "the most difficult to treat," "manage," or "control." (24, 43, 99–102, 108) In addition, a number of articles focused on individuals with histories of homelessness and those considered "hard to house", a target population that was slightly more common in academic articles (45, 58, 79–86, 89–91, 93–95, 109). Six articles discussed programs for forensic mental health patients, or those with justice system involement (34, 103–107). Two studies focused on older adults with SMI discharged from hospital to community residential facilities (73, 74). Among articles that included information on study samples, residents were commonly diagnosed with a schizophrenia spectrum disorder and had a range of support needs. For example, residents of one custodial housing program were assessed as having difficulties meeting basic needs, requiring supervision related to behavior and medication management, and having few personal resources (52). For the target population and sample characteristics of each individual article, see **Supplementary Tables 7** (academic literature) and 8 (grey literature).

### 3.3 High support housing outcomes

Eight outcome domains were examined within the articles included in this review: [1] housing stability; [2] housing satisfaction and preferences; [3] physical and behavioral health; [4] community integration, social support, and quality of life; [5] housing and service costs; [6] offending and recidivism/criminal justice involvement; [7] health service use; and [8] housing supply and demand needs (see **Table 2** and **Supplementary Tables 7**, **8**). Findings from studies with comparison groups are noted, where applicable, though most articles had uncontrolled research designs.

**Table 2:** Outcomes of studies on high support housing.

Authors (year)	Literature type	Housing	Health	Social	Risk and offending	Service access and use	Experience	Costs	None	Other
Anucha (2005) (93)	Academic						X			
Anucha (2006) (94)	Academic						X			
Anucha (2010) (95)	Academic						X			
Aubry & Myner (1996) (46)	Academic			X						
Booth et al. (2023) (47)	Academic					X				
Cherner et al. (2013) (103)	Academic			X			X	X		Program services, goals, and quality
Cherner et al. (2014) (104)	Academic		X	X	X	X	X			
Cochrane et al. (2000) (99)	Academic								X	
Dorvil (1997) (60)	Academic								X	
Dorvil et al. (2005) (62)	Academic								X	
Durbin et al. (2004) (52)	Academic		X							Service/resident needs
Edge & Wilton (2009) (72)	Academic	X		X			X			
Farrell & Aubry (2002) (43)	Academic							X		
Fleury et al. (2010) (110)	Academic									Resident needs; Adequacy of help
Forchuk et al. (2023) (50)	Academic			X			X			
Forchuk et al. (2023) (51)	Academic						X			
Goering et al. (1992) (87)	Academic			X						
Goering et al. (1992) (88)	Academic						X			
Heard et al. (2019) (105)	Academic			X			X			
Kidd et al. (2012) (97)	Academic			X		X		X		Project success
Kirkpatrick & Byrne (2009) (89)	Academic						X			
Kirkpatrick & Byrne (2011) (90)	Academic						X			
Lariviere et al. (2002) (73)	Academic	X	X	X		X				
Lariviere et al. (2006) (74)	Academic	X	X	X						
Lesage et al. (2003) (75)	Academic					X				Housing inventory; Supply/demand
Lesage et al. (2008) (24)	Academic					X		X		
Lesage et al. (2014) (111)	Academic					X		X		Housing inventory; Supply/demand
Nelson et al. (1992) (53)	Academic			X						
Nelson et al. (1997) (54)	Academic	X	X	X			X			Resident empowerment, control
Nelson et al. (1999) (55)	Academic	X		X						Resident empowerment, control
Nelson et al. (2003) (56)	Academic	X		X						
Nelson et al. (2010) (76)	Academic								X	
Newton & Schieldrop (2005) (101)	Academic	X				X		X		Transfer of patients
Patterson et al. (2013) (79)	Academic			X						
Patterson et al. (2014) (80)	Academic			X						
Petersen et al. (2013) (18)	Academic									Transfer of patients
Piat et al. (2002) (63)	Academic	X				X	X	X		
Piat et al. (2004) (64)	Academic			X			X			
Piat et al. (2005) (65)	Academic						X			
Piat et al. (2005) (66)	Academic						X			
Piat et al. (2007) (67)	Academic						X			
Piat et al. (2008) (112)	Academic	X								
Piat et al. (2008) (68)	Academic						X			
Piat et al. (2008) (69)	Academic						X			
Piat et al. (2008) (113)	Academic	X								
Piat et al. (2009) (70)	Academic	X								Resident autonomy
Piat et al. (2011) (44)	Academic						X			
Piat et al. (2015) (71)	Academic	X								
Rezansoff et al. (2017) (81)	Academic		X							
Rudoler et al. (2018) (98)	Academic							X		
Russolillo et al. (2014) (82)	Academic					X				
Somers et al. (2013) (83)	Academic				X					
Somers et al. (2015) (84)	Academic		X							
Somers et al. (2017) (85)	Academic	X	X	X						
Trainor et al. (1993) (78)	Academic									Summarizing past research
Vandevooren et al. (2007) (108)	Academic	X	X			X				
Wasylenki et al. (2000) (102)	Academic								X	
Yamin et al. (2014) (114)	Academic						X			
Addictions and Mental Health Ontario et al. (2018) (109)	Grey	X								
Alberta Health Services (2014) (115)	Grey									Housing inventory; Supply/demand
Butterill et al. (2009) (34)	Grey						X	X		Housing inventory; Supply/demand
Calgary Homeless Foundation (2021) (86)	Grey								X	
Centre for Addiction and Mental Health (2002) (48)	Grey									Housing inventory; Supply/demand
Centre for Addiction and Mental Health (2005) (92)	Grey								X	
Centre for Addiction and Mental Health (2010) (96)	Grey	X	X	X		X	X	X		
Centre for Addiction and Mental Health (2012) (116)	Grey		X	X			X	X		Housing inventory; Supply/demand
Centre for Addiction and Mental Health (2022) (37)	Grey									Housing inventory; Supply/demand
City of Edmonton (n.d.) (117)	Grey									Housing inventory; Supply/demand
Community Support and Research Unit (2012) (49)	Grey	X								Housing inventory; Supply/demand
Dorvil et al. (2003) (61)	Grey	X					X			
Grant & Westheus (2008) (118)	Grey	X	X	X			X			Mastery
Government of Ontario (2017) (119)	Grey									Best practices
High Support Housing Consortium (2009) (120)	Grey							X		Housing inventory; Supply/demand
Homeward Trust Edmonton (2017) (121)	Grey					X				Housing inventory; Supply/demand
Lesage et al. (2006) (100)	Grey	X	X			X				Transfer of patients
Ministry of Mental Health and Addictions (2022) (122)	Grey								X	
Molina et al. (2018) (106)	Grey	X	X	X			X			
Morrow et al. (2006) (123)	Grey									Housing inventory; Supply/demand
Novac & Quance (1998) (57)	Grey									Housing inventory; Supply/demand
Palermo et al. (2006) (124)	Grey							X		
Patterson et al. (2008) (77)	Grey								X	
Sanford et al. (2022) (45)	Grey									Housing inventory; Supply/demand
Serge et al. (2006) (91)	Grey	X	X	X		X				Program evaluation; Service gaps, challenges
SHIP (2014) (125)	Grey								X	
Sirotich et al. (2018) (126)	Grey									Housing inventory; Supply/demand; Resident/applicant characteristics
Suttor (2016) (58)	Grey									Housing inventory; Supply/demand
Trainor (1996) (59)	Grey	X								Program analysis/review
Trainor et al. (2011) (32)	Grey							X		Housing inventory; Supply/demand
Wellesley Institute et al. (2020) (107)	Grey	X								Housing inventory; Supply/demand

#### 3.3.1 Housing stability

Generally, HSH was described as promoting housing stability, and encouraging or preparing residents for more independent accommodations (18, 24, 50, 84, 85, 100, 103–105, 108, 116). For example, in a randomized controlled trial of a single-site HF program, residents spent more time in stable housing (74.3%) than those in treatment as usual (TAU; 27.9%) over 24 months; housing stability of participants assigned to single- and scattered-site HF were comparable (84, 85). In a mixed-methods evaluation of a transitional housing program for 20 forensic mental health patients, 10 in each of two cities, 56% and 27% of residents completed the program and went on to live in their own apartment or elsewhere in the community by 18 months; others continued in the program or were re-hospitalized (104). Elsewhere, a quasi-experimental naturalistic study assessing housing outcomes of 189 patients discharged from long-term psychiatric facilities in British Columbia found that most participants (70.7%) remained housed in HSH settings over a 5-year period, with few transitioning to facilities with less intensive services (18).

#### 3.3.2 Housing satisfaction and preferences

A large number of articles (academic literature: n=26; grey literature: n=8) assessed housing experiences and resident satisfaction, as well as providers' and caregivers' views on residential living situations and accommodation preferences.

Generally, residents of different types of HSH expressed satisfaction with their living conditions, as well as an appreciation of staff presence, relationships with staff and other residents, and provided supports. For example, in a case study of residential care facilities in Hamilton, Ontario, 80% of 50 residents surveyed were "mostly satisfied, satisfied, or delighted" with their current housing overall (72). Similarly, two-thirds of 102 foster home residents surveyed in Quebec expressed a desire to live in their home permanently, with nearly all indicating that they would recommend foster homes to patients discharged from hospital (70).

Literature on both recovery-oriented HSH and alternatives to hospital and other institutions referenced numerous positive aspects to their housing programs. This included a secure and comfortable living environment, as well as available programming and capacity to meet resident needs (50, 51, 87–91, 96, 99, 102–105, 114, 116, 118). Across such settings, residents linked participation in programs to greater self-confidence and sense of independence, skills development and growth, and resiliency (50, 89, 90, 100, 104–106, 114, 116, 118). Similar benefits were also identified by program staff in one report examining multiple HSH sites, with the housing setting providing residents with opportunities to redevelop health and social relationships (116).

Concerns with HSH programs have also been documented. Studies of custodial housing models often described lack of privacy, overcrowding, and diminished resident control (53–55, 59, 64, 72, 76, 78). In board-and-care homes, for example, residents were less likely to have their own room and typically shared facilities with more individuals than those in group homes and supportive apartments (54, 55). Further, board-and-care home residents identified poor food quality, as well as problems with the physical structure and location of their housing (54); similar issues were referenced elsewhere (63, 64, 70, 71). One qualitative study in Quebec highlighted the tensions that foster home residents experienced between "building life around the foster home" and moving on to living elsewhere in the community (68).

Concerns with housing and supports were not limited to historically custodial models. For example, in a multi-methods study investigating facilitators and barriers to housing stability among 106 people considered "hard-to-house," residents of a recovery-oriented HSH program in Toronto, highlighted concerns with the building, program, and staff (93). Echoing findings from research on custodial models, residents expressed a desire for improvements to the building aesthetic and cleanliness (i.e., pest control, landscaping), physical environment (i.e., larger rooms, more private spaces), support and staffing model (i.e., greater support, improved staff training), and safety (i.e., increased building security) (93). Experiences of stigma were also reported (93). In later studies on this same program, residents expressed an appreciation of having their own space and basic housing needs met, but highlighted limited privacy, space constraints, and tensions among program residents (94, 95). Other studies mirrored and extended these findings, with meal plans, housing locations, as well as program rules and restrictions being sources of dissatisfaction among residents (106, 114, 116, 118).

Housing preferences have also been examined in relation to HSH. Studies have highlighted residents' preferences to live in HSH programs rather than hospitals or homeless shelters, as well as a reluctance to leave the alleged security offered by such housing (56, 61, 68, 70, 73, 74). Yet, in several articles, some residents sought a sense of independence beyond was what afforded in HSH, expressing a desire for more autonomous living arrangements (56, 61, 64, 72, 93–96, 112, 113, 116). As for the perspectives of case managers and family members, both groups preferred housing models with close monitoring and supervision, as well as greater clinical involvement for residents (64, 112). For example, in survey research of various supervised housing programs in Quebec, 44% of residents indicated an overall preference for independent apartments, whereas only 11% of case managers shared these views (112).

#### 3.3.3 Physical and behavioral health

Fifteen articles (academic literature: n=9; grey literature: n=6) investigated psychiatric symptoms, severity of disability, functioning, and recovery in the context of HSH, with mixed results. In an analysis of health outcomes in a randomized controlled trial of HF, significant improvements were found in both severity of disability and recovery at 24 months among residents of a single-site HF program compared to TAU (85). Another article from the same trial found no significant differences in medication adherence between the single-site HF and TAU groups (81). Cross-sectional studies examining custodial housing approaches for 33 older adults with SMI reported no significant deterioration or differences in residents' symptomatology, cognitive status, or daily life functioning following discharge from hospital to community residential facilities, regardless of time spent in the community (i.e., 6- to 12-months, 12- to 24-months, ≥24-months) (73, 74). In another retrospective study, reductions in symptoms and improvements in functioning were found among 25 residents participating in a community-based residential treatment and rehabilitation program for people with "complex" needs (108). Other studies have reported improvements in HSH residents' physical health, behavioral health, as well as in ratings of recovery over time, and when compared to other settings (e.g., low-support sites) (54, 91, 96, 100, 104, 116, 118).

Four studies examined rates of substance use among HSH residents (84, 85, 91, 104). No significant differences were found between residents of a single-site HF program and a TAU group in daily substance use after 24 months (84). In a mixed-methods evaluation of a transitional housing program for forensic mental health patients leaving hospital, nine of the 18 individuals who were abstinent from substances at baseline were abstinent at 18 months (104). Finally, in one report detailing several HSH sites and substance use changes, mixed findings were noted (91).

#### 3.3.4 Community integration, social support, and quality of life

Twenty-three articles (academic literature: n=18; grey literature: n=5) reported on residents' community integration, social support and networks, and quality of life, with varying results. Regarding psychological integration, residents of a single-site HF program were more likely to endorse knowing their neighbors, but "not interacting with neighbors or the emotional components of community," compared to participants assigned to the scattered-site HF group (80). Further analysis from the same randomized trial showed that residents of single-site HF experienced significant improvements in psychological integration at 24 months, but no differences on measures of physical integration and quality of life, compared to TAU (85).

Two quasi-experimental studies, a longitudinal study of 107 residents, and a cross-sectional study of 51 residents compared board-and-care homes to other accommodations; no differences in self-reported measures of community integration and quality of life were found between resident groups (46, 54). In contrast, board-and-care home residents reported lower levels of community integration and independent functioning compared to individuals residing in group homes and supportive apartments in another cross-sectional study (53). In qualitative research, residents and caregivers emphasized the importance of integration and relationships within the foster home, whereas professionals valued residents becoming integrated into the broader community (64).

Two studies reported no significant impacts on 33 older adults' perceived quality of life following relocation from psychiatric hospital to custodial HSH. Overall, residents were satisfied with life in the community (73, 74), but social functioning deteriorated among participants living in the community for over two years, controlling for pre-discharge status (74). In an evaluation of a transitional forensic mental health housing program, social support and general life satisfaction among 20 residents remained stable over the first year, with a slight decrease at 18 months (104).

Four studies examined the social networks and support processes of residents, with varying results (53–55, 87). In a cross-sectional study of 42 residents, Goering et al. (87) found a greater number of staff in the social networks of residents provided with 24-hour support; these residents also received more frequent help than residents provided with weekly staff support. Nelson et al. (53) identified no significant differences in network sizes (inclusive of family, friends, and professionals) among 107 residents of board-and-care homes, group homes, and supportive apartment settings. However, residents of group homes and supportive apartments received support from more friends and professionals than residents of board-and-care homes (53). In contrast, later research noted minimal differences in the amount of social support available to residents across these three housing settings, with the authors suggesting that support is not necessarily contingent on housing type (55). Other work by this author found residents of board-and-care homes received more staff support; more emotional and problem-solving support; and less interpersonal conflict and emotional abuse, such as rejection, ridicule, and other forms of verbal abuse, than residents living in supportive apartments (54). Notably, residents in all housing types noted conflicts with their living companions (54).

Positive findings have been reported in the grey literature, with residents of various HSH programs expressing satisfaction with their social supports in one research report (118), and others recognizing increased participation in social and community activities (91, 96, 106, 116).

#### 3.3.5 Housing and service costs

Fourteen articles (academic literature: n=8; grey literature: n=6) reported on housing and service costs associated with HSH. Findings highlighted variation in the costs associated with different types of HSH programs, though lower costs have been recognized as a strength of HSH when compared to inpatient care (32, 34, 43, 96–98, 103, 116, 120). For example, studies reporting on a HSH initiative affiliated with an Ontario psychiatric hospital noted significant cost-savings relative to inpatient care (97, 98), with one analysis estimating savings of $51,000-$58,000 annually, per resident (98). Likewise, in an evaluation of a HSH program's initial year of operation in Ontario, the costs of providing services to patients with delayed discharges from hospital compared favorably to in-patient costs (approximately $76/day in HSH, versus $698/day for a hospital bed) (96). In contrast, in articles reviewing tertiary residential facilities in British Columbia, the mean cost per resident at one facility was $350/day, which was comparable to the per diem cost for the provincial psychiatric hospital (24, 101).

Finally, a simulation study of the number and cost of specialist care facilities across Canada estimated that the costs of supervised group homes, hostels, and foster families were $49,793, $35,616, and $7,746, respectively, per person, per year (111).

#### 3.3.6 Offending and recidivism

Two academic articles reported outcomes related to risk and offending. In an evaluation of a transitional forensic mental health housing program, low rates of re-offending were found; only 15% of residents re-offended within 18 months (104). Further, in a study of a single-site HF program in British Columbia, allocation to the congregate arm was associated with marginally significant reductions in convicted sentences compared to TAU during the study period (83).

#### 3.3.7 Health service use

Fifteen articles (academic literature: n=11; grey literature: n=4) examined transitions out of hospital and health service use, including hospitalizations and emergency department use. In a randomized control trial involving a single-site HF program, residents had non-significant reductions in emergency department use after 12 months compared to TAU (82). Similarly, a cohort study comparing the use of services following the modernization of *Homes for Special Care* to *Community Homes for Opportunity* found a non-significant increase in 368 residents' emergency department use from pre- to post-implementation (47). Rates of primary care service use and specialist care visits increased by 21% and 33%, respectively, over this same period (47).

An evaluation of health service use prior to and after admission into a recovery-oriented HSH program following hospitalization found no significant differences in hospital admission rates post-intervention for 25 participants (108). Other studies on the other hand referenced low rates of re-hospitalization among residents of congregate settings (74, 97). In contrast, many residents of a transitional forensic HSH program required at least one re-hospitalization following admission to the program, with authors noting that the transition to community can be challenging for some residents, and brief re-hospitalization may be appropriate and part of the recovery process (104). These re-hospitalizations were brief, and most residents returned to the community. Lastly, an evaluation of foster home residents in Quebec found reductions in the number of days in hospital, but higher rates of emergency department use post-housing entry; the estimated annual cost of hospital service use was $455,000 pre-housing entry, compared to $86,800 for the first year in housing (63).

Comparable results were described in the grey literature, with findings generally identifying reductions in hospital admissions, emergency department visits, and use of emergency medical services among residents (91, 96, 100, 121). For example, an evaluation of a congregate supportive housing program in Ontario noted the successful transition of residents discharged out of hospital, as well as no hospital readmissions within the first year of operation (96).

#### 3.3.8 Housing supply and demand needs

Eighteen articles (academic literature: n=2; grey literature: n=16) examined the capacity of available HSH programs and level of demand in a region of Canada. These articles outlined critical shortages in supportive housing stock in general and called for more investment in housing for people with SMI, including HSH options (34, 37, 45, 48, 57, 58, 107, 116, 117, 120, 121, 126). According to one assessment, as of 2019, there were approximately 960 individuals waiting for HSH in Toronto, and accommodations for an additional 6,400 individuals in need of 24-hour or daily support would be required over the next 10 years (45). Elsewhere, two academic articles estimated the need for various HSH models in Quebec (75, 111); in one such study, 14 hostels, 26 foster families, and 28 supervised group homes were required per 100,000 inhabitants (111).

Similar concerns and recommendations have been noted regarding specific SMI populations (34, 37, 107, 116). For example, one report found that only 9% of existing supportive housing programs for people with mental illness and justice involvement provided 24-hour supports (107). Further, a five-year projection analysis published in 2019 found that 66–80 additional units of HSH were needed to accommodate patients awaiting discharge from a single Ontario mental health and addictions hospital (37).

## 4 Discussion

This scoping review examined the program characteristics and outcomes of HSH for people with SMI in Canada, drawing from both the academic and grey literature. Notably, there was considerable variation in study designs and reporting, as well as among the HSH programs described in the literature. Prior research has similarly highlighted the heterogeneity of models and practices, as HSH models evolved organically over time without established guidelines (1–4, 127). Despite diversity in program descriptions, there were commonalities between some programs that facilitated the identification of three general types of HSH: [1] custodial HSH; [2] recovery-oriented HSH; and [3] alternatives to hospital programs and other institutions. Yet, these models were nonexclusive and comparisons across studies and types of HSH were infeasible, similar to previous research (4).

Our findings further underscored how HSH programs in Canada evolved over the past several decades, with literature describing the modernization of older custodial housing models (47, 49–51). Recovery-oriented HSH and alternatives to hospital and other institutions, in particular, aim to be responsive to the needs of residents and to promote autonomy. Findings from a single-site HF program in Vancouver, for example, highlighted how evidence-based practices can be adapted into programs offering 24-hour, onsite supports to encourage resident empowerment and choice, generally without producing inferior outcomes compared to scattered-site HF (79–85). As some residents will continue to require 24-hour support, it will be important to continue exploring how best to support residents' basic needs in the least restrictive setting, while also promoting positive living conditions, empowerment, choice, and community integration.

The articles included in this review examined a range of outcomes, including housing stability, physical and behavioral health, quality of life, community integration, and housing satisfaction and preferences. Regarding housing outcomes, HSH programs were generally effective in stably housing residents, with studies incorporating a comparison group that did not involve housing provision documenting superior outcomes for those who received HSH (84, 85). As for health and wellness outcomes, and like the broader research on supportive housing, evidence on HSH was generally mixed (4, 11, 127). This underscores the need for rigorous research on HSH and the development of best practices.

Housing costs, supply, and demand were other types of system-level outcomes examined in the literature. Findings in these domains were more congruous, with most studies associating HSH programs with reduced costs compared to community living and inpatient care; these results generally align with related research on the cost-effectiveness of HF for homeless adults with mental illness (128, 129), underscoring cost offsets associated with housing and support provision. Findings on HSH supply and demand are important to consider in a costing context. Articles in this review highlighted the need for more HSH in various regions of Canada, with some studies providing estimates of the number of programs needed by population size (75, 111). As the demand for supportive housing has grown in many communities across Canada, it is important that a proportion of new stock provide 24-hour, onsite support. A 2010 study from Toronto, Ontario previously estimated that approximately 9% of people experiencing chronic homelessness require 24-hour, onsite supports, and many psychiatric patients experience delayed hospital discharges for lack of HSH; however, updated assessments are required (33, 34).

Finally, our review revealed important findings on the housing and support preferences of HSH residents. These findings highlight both positive and negative aspects of HSH for residents, and diversity in individual preferences. For example, residents were appreciative of program staff presence, relationships, and supports offered. However, residents in several studies expressed a preference for more independent and autonomous living arrangements; these findings are consistent with what is reported in the international literature (3, 12, 13, 127, 130). Residents also identified housing concerns and tensions, underscoring how experiences in HSH may vary, and how support needs and preferences may change over time. Accordingly, there is a need for step-down housing options for those looking to transition on from HSH. As research has shown that Moving On initiatives (i.e., interventions to enable permanent supportive housing residents to move to affordable housing with less intensive supports) yield positive housing outcomes (131), this warrants further examination in the context of HSH for people with SMI.

There are several important limitations to acknowledge. First, due to varying and often limited descriptions of housing models and programs, it was difficult to identify and categorize housing interventions that provided "high" support, as defined in this review. Such a limitation has been noted elsewhere, with previous research on supportive housing finding inconsistencies in the application of service labels (3, 4, 8). Second, no methodological restrictions were placed on the types of articles included. This was intentional for the purpose of comprehensively scoping the literature, but limited comparisons between studies due to methodological and programmatic differences. Third, an operational definition of HSH was developed for this study for which meal preparation and medication management were not requirements (albeit available in most programs), though a more conservative definition might have included these support components. This limitation also stresses the need for definitions of HSH that clearly outline the essential components of the model, and how it differs from other housing interventions for this population.

## 5 Conclusion

People with SMI who are severely disabled have complex and varying needs, capabilities, and goals, often requiring 24-hour, onsite supports. Although safe well-maintained housing is imperative for health and well-being, there is an insufficient supply of HSH in Canada, and no well-established, evidence-based practices. This scoping review demonstrates that research on HSH in Canada is less developed compared to other housing interventions, such as HF and permanent supportive housing. Further longitudinal research on HSH is needed to determine how to optimally advance recovery and social inclusion outcomes in these settings, and how to address evolving resident needs and preferences over time.
