# Supplementary material for: High support housing for people with serious mental illness in Canada: a scoping review
Source: Front Psychiatry. 2025 Jun 24;16:1612516. doi: 10.3389/fpsyt.2025.1612516 (PMC12234495; doi:10.3389/fpsyt.2025.1612516)
Supplement: Supplementary file 1 [file Table1.docx]

**Supplemental Table 1.** Preferred Reporting Items for Systematic reviews and Meta-Analyses extension for Scoping Reviews (PRISMA-ScR) Checklist

| **SECTION** | **ITEM** | **PRISMA-ScR CHECKLIST ITEM** | **REPORTED ON PAGE #** |
| --- | --- | --- | --- |
| **TITLE** | | | |
| Title | 1 | Identify the report as a scoping review. | 1 |
| **ABSTRACT** | | | |
| Structured summary | 2 | Provide a structured summary that includes (as applicable): background, objectives, eligibility criteria, sources of evidence, charting methods, results, and conclusions that relate to the review questions and objectives. | 2-3 |
| **INTRODUCTION** | | | |
| Rationale | 3 | Describe the rationale for the review in the context of what is already known. Explain why the review questions/objectives lend themselves to a scoping review approach. | 4-6 |
| Objectives | 4 | Provide an explicit statement of the questions and objectives being addressed with reference to their key elements (e.g., population or participants, concepts, and context) or other relevant key elements used to conceptualize the review questions and/or objectives. | 6 |
| **METHODS** | | | |
| Protocol and registration | 5 | Indicate whether a review protocol exists; state if and where it can be accessed (e.g., a Web address); and if available, provide registration information, including the registration number. | 6 |
| Eligibility criteria | 6 | Specify characteristics of the sources of evidence used as eligibility criteria (e.g., years considered, language, and publication status), and provide a rationale. | 6-8 |
| Information sources* | 7 | Describe all information sources in the search (e.g., databases with dates of coverage and contact with authors to identify additional sources), as well as the date the most recent search was executed. | 8-9 |
| Search | 8 | Present the full electronic search strategy for at least 1 database, including any limits used, such that it could be repeated. | Supplemental Materials |
| Selection of sources of evidence† | 9 | State the process for selecting sources of evidence (i.e., screening and eligibility) included in the scoping review. | 8-9 |
| Data charting process‡ | 10 | Describe the methods of charting data from the included sources of evidence (e.g., calibrated forms or forms that have been tested by the team before their use, and whether data charting was done independently or in duplicate) and any processes for obtaining and confirming data from investigators. | 9 |
| Data items | 11 | List and define all variables for which data were sought and any assumptions and simplifications made. | 9 |
| Critical appraisal of individual sources of evidence§ | 12 | If done, provide a rationale for conducting a critical appraisal of included sources of evidence; describe the methods used and how this information was used in any data synthesis (if appropriate). | 9 |
| Synthesis of results | 13 | Describe the methods of handling and summarizing the data that were charted. | 9 |
| **RESULTS** | | | |
| Selection of sources of evidence | 14 | Give numbers of sources of evidence screened, assessed for eligibility, and included in the review, with reasons for exclusions at each stage, ideally using a flow diagram. | 9-10 & Figure 1 |
| Characteristics of sources of evidence | 15 | For each source of evidence, present characteristics for which data were charted and provide the citations. | 10 & Supplemental Materials |
| Critical appraisal within sources of evidence | 16 | If done, present data on critical appraisal of included sources of evidence (see item 12). | N/A |
| Results of individual sources of evidence | 17 | For each included source of evidence, present the relevant data that were charted that relate to the review questions and objectives. | Table 1 & Supplemental Materials |
| Synthesis of results | 18 | Summarize and/or present the charting results as they relate to the review questions and objectives. | 10-22 |
| **DISCUSSION** | | | |
| Summary of evidence | 19 | Summarize the main results (including an overview of concepts, themes, and types of evidence available), link to the review questions and objectives, and consider the relevance to key groups. | 22-25 |
| Limitations | 20 | Discuss the limitations of the scoping review process. | 24-25 |
| Conclusions | 21 | Provide a general interpretation of the results with respect to the review questions and objectives, as well as potential implications and/or next steps. | 25 |
| **FUNDING** | | | |
| Funding | 22 | Describe sources of funding for the included sources of evidence, as well as sources of funding for the scoping review. Describe the role of the funders of the scoping review. | 27 |

**Supplemental Table 2.** Ovid Embase Search Strategy

| Embase Classic+Embase <1947 to 2023 July 18> | | |
| --- | --- | --- |
| 1 | mental disease/ | 302119 |
| 2 | ((complex or severe* or serious* or persist* or chronic* or significant*) adj (mental* or psychiatr* or psycholog*)).ti,ab,kf. | 42968 |
| 3 | homeless person/ | 3026 |
| 4 | (ill-housed or unhoused or street people or street dwelling or shelter* or homeless* or forensic*).ti,ab,kf. | 112391 |
| 5 | (alternat* level* of care or alternat* care).ti,ab,kf. | 1240 |
| 6 | or/1-5 | 433996 |
| 7 | tertiary health care/ | 47609 |
| 8 | residential care/ or residential home/ | 21736 |
| 9 | housing/ | 33681 |
| 10 | assisted living facility/ | 3184 |
| 11 | halfway house/ | 1614 |
| 12 | ((support* or permanent or resident* or assisted or custodial or group or communit*) adj2 (hous* or home* or accommodation* or service* or unit* or facilit* or living*)).ti,ab,kf. | 128185 |
| 13 | (hous* adj2 (program* or intervention* or service* or model*)).ti,ab,kf. | 6226 |
| 14 | or/7-13 | 226917 |
| 15 | 6 and 14 | 14888 |

**Supplemental Table 3.** PsycInfo Search Strategy

| APA PsycInfo <1806 to July Week 2 2023> | | |
| --- | --- | --- |
| 1 | mental disorders/ or chronic mental illness/ or serious mental illness/ | 99480 |
| 2 | ((complex or severe* or serious* or persist* or chronic* or significant*) adj (mental* or psychiatr* or psycholog*)).ti,ab,id. | 31824 |
| 3 | homeless/ or homeless mentally ill/ | 9028 |
| 4 | (ill-housed or unhoused or street people or street dwelling or shelter* or homeless* or forensic*).ti,ab,id. | 41463 |
| 5 | (alternat* level* of care or alternat* care).ti,ab,id. | 393 |
| 6 | or/1-5 | 154500 |
| 7 | residential care institutions/ or halfway houses/ or assisted living/ or group homes/ | 13448 |
| 8 | Housing/ | 6615 |
| 9 | ((support* or permanent or resident* or assisted or custodial or group or communit*) adj2 (hous* or home* or accommodation* or service* or unit* or facilit* or living*)).ti,ab,id. | 64706 |
| 10 | (hous* adj2 (program* or intervention* or service* or model*)).ti,ab,id. | 2766 |
| 11 | or/7-10 | 78386 |
| 12 | 6 and 11 | 8853 |

**Supplemental Table 4.** CINAHL Search Strategy

| # | Search Terms | Search Options | Results |
| --- | --- | --- | --- |
| S1 | (MH "Mental Disorders") | **Search modes** - Boolean/Phrase | 66,177 |
| S2 | (MH "Mental Disorders, Chronic") | **Search modes** - Boolean/Phrase | 2,083 |
| S3 | TI ( ((complex or severe* or serious* or persist* or chronic* or significant*) N1 (mental* or psychiatr* or psycholog*)) ) OR AB ( ((complex or severe* or serious* or persist* or chronic* or significant*) N1 (mental* or psychiatr* or psycholog*)) ) | **Search modes** - Boolean/Phrase | 17,023 |
| S4 | (MH "Homeless Persons") OR (MH "Homelessness") | **Search modes** - Boolean/Phrase | 10,480 |
| S5 | TI ( (ill-housed or unhoused or street people or street dwelling or shelter* or homeless* or forensic*) ) OR AB ( (ill-housed or unhoused or street people or street dwelling or shelter* or homeless* or forensic*) ) | **Search modes** - Boolean/Phrase | 25,511 |
| S6 | TI ( (alternat* level* of care or alternat* care) ) OR AB ( (alternat* level* of care or alternat* care) ) | **Search modes** - Boolean/Phrase | 626 |
| S7 | S1 OR S2 OR S3 OR S4 OR S5 OR S6 | **Search modes** - Boolean/Phrase | 104,654 |
| S8 | (MH "Tertiary Health Care") | **Search modes** - Boolean/Phrase | 11,009 |
| S9 | (MH "Residential Care") OR (MH "Residential Facilities") OR (MH "Assisted Living") | **Search modes** - Boolean/Phrase | 14,707 |
| S10 | (MH "Housing") | **Search modes** - Boolean/Phrase | 9,693 |
| S11 | TI ( ((support* or permanent or resident* or assisted or custodial or group or communit*) N2 (hous* or home* or accommodation* or service* or unit* or facilit* or living*)) ) OR AB ( ((support* or permanent or resident* or assisted or custodial or group or communit*) N2 (hous* or home* or accommodation* or service* or unit* or facilit* or living*)) ) | **Search modes** - Boolean/Phrase | 88,388 |
| S12 | TI ( (hous* N2 (program* or intervention* or service* or model*)) ) OR AB ( (hous* N2 (program* or intervention* or service* or model*)) ) | **Search modes** - Boolean/Phrase | 3,672 |
| S13 | S8 OR S9 OR S10 OR S11 OR S12 | **Search modes** - Boolean/Phrase | 118,146 |
| S14 | S7 AND S13 | **Search modes** - Boolean/Phrase **Limiters -** Published Date: -20230731 (July 2023) | 8,835 |

**Supplemental Table 5.** Description of Included Articles (Academic Literature)

| **Author (Year)** | **Region of Canada** | **Article Objective** | **Article/Study Type** |
| --- | --- | --- | --- |
| Anucha (2005)^92^ | Toronto, ON | To explore the housing, neighbourhood, and community needs of formerly homeless people living in two housing programs in Toronto | Cross-sectional study |
| Anucha (2006)^93^ | Toronto, ON | To examine the experiences of Strachan House staff in implementing an alternative housing philosophy within a communal setting | Case study |
| Anucha (2010)^94^ | Toronto, ON | To discuss the challenges associated with shared housing models for rehousing people who are considered “hard to house” | Qualitative study |
| Aubry & Myner (1996)^45^ | Ottawa-Carleton, ON | To compare community integration and quality of life among people with psychiatric disabilities living in housing programs to community residents | Cross-sectional study |
| Booth et al. (2023)^46^ | ON | To compare the use of healthcare services of people with severe and persistent mental illness, before and after the modernization of Homes for Special Care to Community Homes for Opportunity | Cohort study |
| Cherner et al. (2013)^102^ | ON | To evaluate the implementation of the Transitional Rehabilitation Housing Pilot program in two cities | Mixed-methods program evaluation |
| Cherner et al. (2014)^103^ | ON | To describe the outcomes of the Transitional Rehabilitation Housing Pilot program for forensic patients transitioning to the community | Mixed-methods program evaluation |
| Cochrane et al. (2000)^98^ | N/A | To describe tertiary care subpopulations, provide prevalence estimates, and discuss best practices for addressing their special needs | Overview |
| Dorvil (1997)^59^ | QC | To describe the process of transitioning mental health services from hospitals to the community, as well as provide examples of housing models, with a focus on Quebec | Qualitative study |
| Dorvil et al. (2005)^61^ | QC | To examine perceptions of quality of life and degree of empowerment among people using mental health services in relation to different types of housing (residential accommodations, apartment-type resources), with a focus on Quebec | Overview |
| Durbin et al. (2004)^51^ | ON | To examine the impairment and service need levels of a subgroup of residents of the Homes for Special Care program | Cross-sectional study |
| Edge & Wilton (2009)^71^ | Hamilton, ON | To examine the extent to which residential care facilities can be “reengineered” to improve residents’ daily lives | Case study |
| Farrell & Aubry (2002)^42^ | N/A | To describe the methods and results of a comprehensive costing approach for assessing the costs of supporting one person with severe and persistent mental illness to live in the community | Case study |
| Fleury et al. (2010)^124^ | Montreal, QC | To describe the needs of consumers with severe mental disorders and the correlates of adequacy of help provided in seven different types of housing | Cross-sectional study |
| Forchuk et al. (2023)^49^ | ON | To examine residents’ experiences of their home environments following the modernization of Homes for Special Care to Community Homes for Opportunity | Qualitative study |
| Forchuk et al. (2023)^50^ | ON | To explore homeowners’ perspectives on the Community Homes for Opportunity program, and related policies and practices | Qualitative study |
| Goering et al. (1992)^86^ | Toronto, ON | To describe and assess the social support networks of residents living in supportive housing operated by two community organizations | Cross-sectional study |
| Goering et al. (1992)^87^ | Toronto, ON | To evaluate the impact and outcomes of a supportive housing organization, Regeneration House Inc. | Program evaluation |
| Heard et al. (2019)^104^ | London, ON | To examine forensic mental health consumers’ experiences in mental health and justice housing | Qualitative study |
| Kidd et al. (2012)^96^ | Toronto, ON | To describe the components and outcomes of a high support housing initiative launched within an Ontario mental health and addictions hospital | Letter to the editor |
| Kirkpatrick & Byrne (2009)^88^ | ON | To explore the experiences of “moving on” from homelessness among individuals with major mental illnesses living in the HOMES (Housing with Outreach, Mobile and Engagement Services) program | Qualitative study |
| Kirkpatrick & Byrne (2011)^89^ | ON | To describe the program narrative of the HOMES (Housing with Outreach, Mobile and Engagement Services) program | Qualitative study |
| Lariviere et al. (2002)^72^ | Montreal, QC | To examine the global functioning, performance, and quality of life in older adults with severe and chronic mental illness, following discharge from a psychiatric hospital to community residential facilities | Longitudinal study |
| Lariviere et al. (2006)^73^ | Montreal QC | To examine levels of adjustment to community life over time in older adults with serious mental illness, following discharge from psychiatric hospital to community residential facilities | Cross-sectional study |
| Lesage et al. (2003)^74^ | Montreal, QC | To describe the use of and projected needs for residential tertiary care in east-end Montreal and compare the results to other Canadian provinces and other countries, with a view to establish national benchmarks | Epidemiology study using two surveys |
| Lesage et al. (2008)^25^ | QC and BC | To review the characteristics of community-based psychiatric residential facilities as an alternative to long-term psychiatric hospitalization | Conceptual article |
| Lesage (2014)^115^ | N/A | To recommend that regional psychiatric rehabilitation centres and authorities be developed to provide psychiatric services, and evidence-based rehabilitation interventions to all people requiring it in a region or province | Commentary |
| Nelson et al. (1992)^52^ | ON | To examine self-reported social network transactions of former psychiatric inpatients residing in different types of supportive housing (supportive apartments, group homes, board-and-care homes) | Cross-sectional study |
| Nelson et al. (1997)^53^ | ON | To examine, describe, and interpret the process and outcomes of three types of housing (supportive apartments, group homes, board-and-care homes) for people with severe and long-term psychiatric disabilities | Longitudinal study with a non-equivalent comparison group |
| Nelson et al. (1999)^54^ | ON | To compare the housing qualities, social support, personal empowerment, and emotional well-being of people with psychiatric disabilities living in different types of housing (supportive apartments, group homes, board-and-care homes) | Cross-sectional study |
| Nelson et al. (2003)^55^ | London, ON | To examine the housing preferences and satisfaction of psychiatric consumers/survivors | Cross-sectional study, with some qualitative data collection |
| Nelson et al. (2010)^75^ | N/A | To describe and analyze the evolution of housing approaches for people with serious mental illness, making distinctions between three housing models (custodial, supportive, supported) | Overview |
| Newton & Schieldrop  (2005)^100^ | BC | To describe a community-based tertiary mental health facility (Seven Oaks) that was developed following the downsizing and replacement of a large provincial mental health hospital | Program description |
| Patterson et al. (2013)^78^ | Vancouver, BC | To examine changes in subjective quality of life among homeless adults with mental illness after random assignment to Housing First (HF) or treatment as usual (TAU), including congregate HF | Randomized controlled trial |
| Patterson et al. (2014)^79^ | Vancouver, BC | To examine community integration among HF residents at 6 and 12 months, compared to TAU | Randomized controlled trial |
| Petersen et al. (2013)^19^ | Vancouver, BC | To assess the clinical and social outcomes of a cohort of patients who transitioned from hospital into Tertiary Psychiatric Residential Facilities | Quasi-experiment |
| Piat et al. (2002)^62^ | Montreal, QC | To evaluate the integration of a nursing assistant into a foster home for persons with a severe mental illness | Program evaluation |
| Piat et al. (2004)^63^ | Montreal, QC | To identify stakeholder perspectives on the dimensions of psychiatric foster homes | Qualitative study |
| Piat et al. (2005)^64^ | Montreal, QC | To examine foster home caregivers’ views on the transformation of mental health services, including the impacts on their work with persons with serious mental illness | Qualitative study |
| Piat et al. (2005)^65^ | Montreal, QC | To explore the nature of the partnership between managers/supervisors of residential care homes and social services networks, with a focus on Quebec | Qualitative study |
| Piat et al. (2007)^66^ | Montreal, QC | To examine caregiver perspectives on the values and qualities required to support people living in foster homes | Qualitative study |
| Piat et al. (2008)^112^ | Montreal, QC | To compare the housing preferences of residents with serious mental illness living in seven different types of housing to those of their case managers | Cross-sectional study |
| Piat et al. (2008)^67^ | Montreal, QC | To describe the housing-related needs of psychiatric foster home residents | Qualitative study |
| Piat et al. (2008)^68^ | Montreal, QC | To explore the perspectives of caregivers on the nature and type of help offered in Montreal foster homes | Qualitative study |
| Piat et al. (2008)^113^ | Montreal, QC | To explore the housing preferences of people with serious mental illness living in seven different types of housing | Qualitative study |
| Piat et al. (2009)^69^ | Montreal, QC | To examine foster home residents’ experiences about their lives and caregivers’ perceptions of permitted autonomy in the residences | Cross-sectional study |
| Piat et al. (2011)^43^ | Montreal, QC | To describe the role of family in supporting recovery for mental health consumers living in structured, community housing | Qualitative study |
| Piat et al. (2015)^70^ | Montreal, QC | To explore the perceptions of residents and housing proprietors on the recovery orientation of structured congregate housing for persons with serious mental illness | Cross-sectional study |
| Rezansoff et al. (2017)^80^ | Vancouver, BC | To evaluate the effects of HF on adherence to antipsychotic medication among homeless adults with schizophrenia | Randomized controlled trial |
| Rudoler et al. (2018)^97^ | Toronto, ON | To conduct a cost analysis of a supportive housing intervention, with comparison to inpatient care for people with severe mental illness designated as ALC | Cost analysis |
| Russolillo et al. (2014)^81^ | Vancouver, BC | To study the effectiveness of HF as means of reducing emergency department utilization among participants randomised to congregate HF, scattered-site HF, or TAU | Randomized controlled trial |
| Somers et al. (2013)^82^ | Vancouver, BC | To examine the effect of HF on criminal behaviour among individuals experiencing homelessness and mental illness, who had previous involvement with the justice system | Randomized controlled trial |
| Somers et al. (2015)^83^ | Vancouver, BC | To compare daily substance use between HF and TAU over 12 and 24 months | Randomized controlled trial |
| Somers et al. (2017)^84^ | Vancouver, BC | To investigate the effectiveness of HF in both scattered site and congregate formats, compared to TAU, on housing, health, and psychosocial outcomes over 24 months | Randomized controlled trial |
| Trainor et al. (1993)^77^ | N/A | To compare two types of community housing models for individuals with mental illnesses (custodial housing and alternative housing models) and discuss key issues in alternative housing development | Overview |
| Vandevooren et al. (2007)^107^ | N/A | To evaluate the outcomes of the community-based Steele Street Residential Treatment and Rehabilitation Program | Within-subjects study |
| Wasylenki et al. (2000)^101^ | N/A | To define and describe tertiary care, including consumer profiles, program elements, tertiary care program models (e.g., residential care), systems of care, and planning targets | Overview |
| Yamin et al. (2014)^111^ | Moncton, NB | To describe a supportive housing pilot program (Peer Supportive Housing) developed at the Moncton site of the At Home/Chez Soi demonstration project | Qualitative study |

**Note**. ON: Ontario; QC: Quebec; BC: British Columbia; NB: New Brunswick; HF: Housing First; ALC: Alternate Level of Care; TAU: Treatment as Usual; N/A: Not Applicable

**Supplementary Table 6.** Description of Included Articles (Grey Literature)

| **Author (Year)** | **Region of Canada** | **Article Objective** | **Article/Study Type** |
| --- | --- | --- | --- |
| Addictions and Mental Health Ontario et al. (2018)^108^ | ON | To document examples of practices in supportive housing for people with mental health and addiction issues, with a focus on Ontario | Resource guide |
| Alberta Health Services (2014)^125^ | AB | To establish a framework outlining supportive housing, treatment and care options, provider roles, and funding accountabilities | Framework |
| Butterill et al. (2009)^33^ | ON | To identify the clinical characteristics of ALC/long-stay patients, as well as the housing, community services, and supports required to transition them into the community | Multi-methods study |
| Calgary Homeless Foundation (2021)^85^ | AB | To serve as a resource guide for housing programs | Practice guide |
| Centre for Addiction and Mental Health (2002)^47^ | Toronto, ON | To point critical issues in housing for people with mental illness, and to suggest new approaches | Discussion paper |
| Centre for Addiction and Mental Health (2005)^91^ | Toronto, ON | To outline housing options and resources available for individuals receiving mental health and substance use services; designed to aid workers, clients, and their families in finding suitable housing within the Toronto community | Guide |
| Centre for Addiction and Mental Health (2010)^95^ | Toronto, ON | To evaluate the process and outcomes of the first year of operation (August 2009-July 2010) of 90 Shuter St., offering high support services to long-stay alternative level of care (ALC) clients in the Centre for Addiction and Mental Health (CAMH) Schizophrenia Program | Report (draft) |
| Centre for Addiction and Mental Health (2012)^109^ | Toronto, ON | To explore clients’ perceptions of supportive housing, and the transition from hospital to community | Qualitative study |
| Centre for Addiction and Mental Health (2022)^36^ | ON | To provide a model for housing and mental health policies that addresses the needs of people with mental illness and those with substance use disorders | Policy report |
| City of Edmonton (n.d.)^117^ | Edmonton, AB | To outline community intentions in response to priority housing and support needs, with a focus on the Edmonton area | Community plan |
| Community Support and Research Unit (2012)^48^ | n/a | To review the evolution of and practices in custodial housing, and to provide support for the transition away from this model of housing in Canada | Position paper |
| Dorvil et al. (2003)^60^ | QC | To explore housing options for individuals with mental disorders by comparing three types of housing (natural family, foster homes, social housing with community support); documents residents’ perceptions of housing preferences, support provision, and progress towards recovery | Research report |
| Grant & Westheus (2008)^110^ | Toronto, ON | To compare outcomes of two housing models (high support, low support) for individuals with serious mental illness, including program satisfaction and preferences | Research report |
| Government of Ontario (2017)^128^ | ON | To provide information on best practices in supportive housing and related services/systems | Practice guide |
| High Support Housing Consortium (2009)^114^ | Toronto, ON | To outline findings from a survey circulated among high-support housing providers and agencies that refer individuals to these providers (“referral sources”) | Report (draft) |
| Homeward Trust Edmonton (2017)^116^ | AB | To describe the strengths, opportunities, and gaps in Edmonton’s permanent supportive housing sector | Report |
| Lesage et al. (2006)^99^ | BC | To compare patients admitted to Seven Oaks and South Hills with those receiving treatment at Riverview Hospital | Report (draft) |
| Ministry of Mental Health and Addictions (2022)^127^ | BC | To outline key elements for delivering consistent, high-quality services tailored to local needs, aiming to address underserved populations, with a focus on the development of Complex Care Housing | Strategic framework |
| Molina et al. (2018)^105^ | Toronto, ON | To outline findings of an evaluation of the Transitional Rehabilitation Housing Program (TRHP 1); evaluation examines how TRHP 1 contributes to the psychosocial rehabilitation and recovery of its participants, along with their integration into the community and transition into mainstream mental health services | Report (draft) |
| Morrow et al. (2006)^126^ | BC | To describe existing services, recent funding and policy changes, and the broader impact of policy and service delivery changes on income security and housing for individuals with mental illness | Research report |
| Novac & Quance (1998)^56^ | Toronto, ON | To provide an overview of supportive housing, with a focus on Toronto, Ontario | Report |
| Palermo et al. (2006)^130^ | Halifax, NS | To examine homelessness and evaluate costs and advantages of implementing supportive housing in Metro Halifax | Report |
| Patterson et al. (2008)^76^ | BC | To describe the population of individuals experiencing homelessness or inadequate housing who have severe addictions and/or mental illness, including recommended solutions and associated costs | Report |
| Sanford et al. (2022)^44^ | Toronto, ON | To conduct a needs assessment of mental health and addictions supportive housing, including challenges experiences by service users and providers, estimated number of needed supportive housing units, and strategies to address existing housing shortfalls | Assessment |
| Serge et al. (2006)^90^ | Canada | To investigate service approaches for people with concurrent disorders who are homeless or at risk of homelessness | Report |
| SHIP (2014)^129^ | ON | To report on SHIP’s model and services, including successes, direction, and development of the organization | Annual report |
| Sirotich et al. (2018)^118^ | Toronto, ON | To examine the characteristics of supportive housing applicants, their support needs, housing preferences, wait lists, and outcomes | Report |
| Suttor (2016)^57^ | ON | To report on supportive housing approaches for people living with mental illness, addiction, or chronic homelessness | Report |
| Trainor (1996)^58^ | Toronto, ON | To explore integrating the Homes for Special Care program into the mental health reform efforts, involving consultations with stakeholders, reviewing relevant literature, and seeking legal advice to inform the process | Analysis (draft) |
| Trainor et al. (2011)^31^ | Canada | To assess the current status of and needs regarding housing and support for individuals with mental health issues; seeks to identify exemplary housing programs, determine essential community services, and assess the overall costs and benefits of providing specialized housing and support services | Report |
| Wellesley Institute et al. (2020)^106^ | Toronto, ON | To assess the needs of justice-focused mental health and addictions supportive housing and propose recommendations for action | Report |

**Note**. AB: Alberta; ON: Ontario; BC: British Columbia; QC: Quebec; ALC: Alternate Level of Care; CAMH: Centre for Addiction and Mental Health; SHIP: Supportive Housing in Peel; TRHP Transitional Rehabilitation Housing Program

**Supplemental Table 7**. Description of High Support Housing Program and Outcomes (Academic Literature)

| **Authors (Year)** | **Description of Housing Model/Program** | **Objectives/Values of Housing Program** | **Target Program Population & Resident Characteristics** | **Key Findings** |
| --- | --- | --- | --- | --- |
| Anucha (2005, 2006, 2010)^92-94^ | Anucha (2006)^93^   - *Strachan House*, developed by the Homes First Society and in collaboration with community partners and residents, provides housing for approximately 70 formerly homeless people - Residents live in private, individual units; some spaces are shared (bathroom, kitchen, living area) - Community workers are onsite 24/7 and each staff member is assigned to a “house” consisting of 5-8 resident rooms linked to shared living spaces - Staff help residents access available supports and facilitate house meetings   Anucha (2005, 2010)^92,94^   - Two alternative housing programs (*Strachan House, Keith Whitney Centre*) provide housing to ~260 clients in three buildings operated by two agencies; programs are comparable in structure and mandates | Anucha (2005, 2006, 2010)^92-94^   - Facilitative management approach grounded in community development values and principles | Anucha (2005, 2006, 2010)^92-94^  Target program population:   - People with histories of homelessness who are characterized as “hard to house”   Anucha (2005)^92^  Characteristics only provided for sample of residents (N=106):   - Mean age: ~45 years - Gender: M=60.4% - Mean number of lifetime homelessness episodes: ~4   Anucha (2006)^93^  Not described  Anucha (2010)^94^  Characteristics only provided for sample of residents (N=12):   - Age range: 28-57 years - Gender: F=8, M=4 - Behavioural health: 8 had substance use problems, 1 had a serious mental illness, 1 had physical health problems - Housing history: 11 had multiple episodes of homelessness | Anucha (2005)^92^   - To maintain stability, residents suggested aesthetic and cleanliness improvements to housing (e.g., painting, pest control), physical improvements (e.g., bigger rooms, more privacy), and staffing/program improvements (e.g., greater staff support, effectively trained staff) - Residents highlighted the importance of matching individuals to appropriate units - Issues related to crime, sex work, general security, and safety within the house and neighbourhood were identified; residents wanted increased protection and patrols - Experiences of stigma were described in relation to the housing, and feeling unable to contribute within the community - Three types of housing were recommended by residents: subsidized, public and/or affordable housing; shared housing with staff support; special needs housing   Anucha (2006)^93^   - Working within an empowerment framework was perceived to be necessary to give residents a voice and contribute to a “sense of belonging,” according to community housing workers - 24/7 onsite staff presence, close staff contact, and programming (e.g., excursions, meal programs, tenant bank, employment programs) were viewed as valuable in supporting residents and in maintaining housing   Anucha (2010)^94^   - Residents valued having their own space and basic housing needs met within shared housing; challenges were identified, such that housing did not feel like “home” and residents felt like they were “in some respects still homeless,” attributed, in part, to limited privacy, communal spaces, and tensions among residents - Residents expressed that their housing prevented them from pursuing relevant goals (e.g., seeing family, finding employment) |
| Aubry & Myner (1996)^45^ | - Board-and-care residences are congregate living settings, where shelter, meals, and supervision, but no formal rehabilitation are provided; other housing programs offering supports and rehabilitation, in addition, to shelter, meals, and supervision, were also included in the study - The number of residents per housing program ranged from 9 to 124 | Not described | Target program population:   - Persons with psychiatric disabilities   Characteristics only provided for sample of persons with psychiatric disabilities (N=51):   - Behavioural health: 57% had schizophrenia; 14% had affective disorders - Housing: 39 persons lived in board-and-care residences; 12 persons lived in other housing programs | - Persons with psychiatric disabilities residing in board-and-care homes did not differ from those living in supportive housing programs on measures of community integration and quality of life |
| Booth et al. (2023)^46^  Forchuk et al. (2023)^49,50^ | *Homes for Special Care* (HSC):   - Long-term, single-site (group home) permanent supportive housing offered to people from provincial psychiatric hospitals; managed through the hospital’s psychiatric system - Staff, meals, supervision, and assistance with daily living available 24/7 - Services are conditional on staying within the program   *Community Homes for Opportunity* (CHO):   - A modified version of HSC, providing individuals with access to subsidized group home settings with 24-hour supports - Residents are provided with financial support and assistance with life skills through various activities and programming (e.g., furthering education, financial literacy, personal hygiene, resource management, leisure activities) - Services are offered both within the home and in the community - Professional staff promote resident growth and independence; residents are paired with a community social worker - Residents given a voice in housing decision, such as choosing where to live and lease signing | HSC:   - Originally seen as an alternative to institutional psychiatric care in Ontario - Criticized for limiting resident autonomy and individual choice   CHO:   - Utilizes a person-centred, community-based housing approach to promote resident recovery, autonomy, and community integration - Empowers residents to be active participants in their goal-setting, planning, and individual choices | Booth et al. (2023)^46^, Forchuk et al. (2023)^49,50^  Target program population:   - Persons with mental illness   Booth et al. (2023)^46^  Characteristics only provided for sample of HSC residents (N=368):   - Mean age: 56.5 years - Gender: F=143 - Mean duration of stay in HSC: 73.48 months - Behavioural health: 336 had psychotic disorders, 269 had non-psychotic disorders (i.e., mood disorders), 79 had addiction and substance use diagnoses, and 89 had other mental illnesses   Forchuk et al. (2023)^49^  Characteristics only provided for sample of residents (N=188):   - Age range: 23-79 years - Gender: F=42% - Behavioural health: ~95% of the sample had at least one psychiatric diagnosis (schizophrenia, mood disorders, anxiety disorders, and substance-related disorder were most common)   Forchuk et al. (2023)^50^  Not described | Booth et al. (2023)^46^   - Across the pre-, peri-, and post-implementation periods, primary health care and specialist resources were most regularly used by residents - Residents who transitioned to CHO used more health services; A 21% increase in the use in primary health care services and a 33% increase in specialist care visits was found in the post-implementation period, compared to the pre-implementation period - Emergency department use non-significantly increased over the post-implementation period   Forchuk et al. (2023)^49^   - Modernization of the HSC program to CHO was seen as an improvement by residents; residents were generally satisfied with CHO staff, services, and program supports, with medium- and long-term benefits described - Residents expressed that CHO fostered autonomy and independence, including in managing one’s own resources and opportunities to further education - Residents reported that CHO promoted interpersonal relationships, including with neighbours; CHO residents’ social and community involvement, and quality of life, improved in the program   Forchuk et al. (2023)^50^   - Homeowners expressed positive feelings about CHO program implementation, and the supports and compensation available to them; CHO staff mentioned developing good, healthy relationships with residents - Homeowners asserted that the CHO program offered residents opportunities to be involved in the community and to engage in social activities, provided greater financial assistance and supports, and improved residents’ standard of health, comfort, and happiness - Compared to HSC, homeowners perceived CHO as more adaptable to the needs of residents, fostering individuality and independence |
| Cherner et al. (2013, 2014)^102,103^ | - Two transitional housing programs (City A, City B), each serving forensic mental health patients, are described; each offers residences with 24-hour staff supervision - Residents are provided with opportunities to participate in recovery-oriented skills training (e.g., cooking, laundry) and social events, intended to improve living skills and facilitate community integration - Onsite staff included transitional support workers or case managers, as well as nursing and psychiatric supports - Staff provided individualized supports tailored to residents’ needs, including goal-setting, counselling, and crisis intervention; group programming is offered | To transition forensic patients successfully into the community using a psychosocial rehabilitation approach | Target program population:   - Individuals declared Not Criminally Responsible of an illegal act on account of mental disorder and being discharged from hospital - Clients under the purview of the Ontario Review Board   Cherner et al. (2013)^102^  Characteristics only provided for sample (N=20):   - Mean age: ~33 years - Gender: M=18, F=2 - Behavioural health: 18 had a psychotic disorder, 3 had a mood or anxiety disorder, 8 had a personality disorder, and 12 a substance use disorder   Cherner et al. (2014)^103^  Characteristics only provided for sample (N=20):   - Mean age: approximately 33 years - Gender: M=18, F=2 - Behavioural health: 18 had schizophrenia, 13 had a substance use disorder, 8 had a personality disorder, 2 had a mood or anxiety disorder, 1 had a developmental disability | Cherner et al. (2013)^102^   - Overall, the program was being implemented as planned and serving the intended population - Program managers, staff, and residents commented on the cleanliness, tidiness, and furnishing of the housing units; Conditions of the housing created a sense of comfort and security - Residents expressed interest in the social activities offered; Residents appeared to receive less support in terms of education and work, though interested in pursuing further education and in obtaining employment - In terms of supports and supervision, the delivery of the program corresponded well with planned implementation; there was greater focus on community integration, and less on basic skill development, than anticipated - Program strengths were identified by community and hospital partners, key informants, and residents, including: facilitating growth and recovery among residents, offering opportunities to adopt “normal roles” in the community; reduced costs, compared to hospital inpatient stay; the openness and availability of staff, contributing to a strong therapeutic relationship; program was perceived as being “client-centered”   Cherner et al. (2014)^103^   - Of the 18 residents who abstained from substances at baseline, nine were abstinent at 18 months - Community ability/functioning was stable over time; social support, recovery, and life satisfaction were stable over time, with a slight decrease at 18 months - 55% were re-hospitalized and 15% re-offended within the 18 months - By end of data collection, 56% of City A and 27% of City B residents completed the program and were living in their own apartment of in the community - Residents valued aspects of programming, such as recreation, substance use groups, and cognitive behavioural therapy |
| Cochrane et al. (2000)^98^  Wasylenki et al. (2000)^101^ | - Tertiary residential programs are a housing and support model developed to provide safe environments and to address the special needs of tertiary care subpopulations, such as individuals with concurrent disorders; examples are provided (e.g., *The Seven Oaks Project*), offering an alternative to hospital-based care - Trained staff are involved in daily care delivery; residents have access to inpatient services, respite care, and consultation/education - Programs are ideally located in small settings, with high staff-to-resident ratios - Tertiary residential programs may offer recreational, vocational, and life skills training opportunities for residents through partnerships with specialized day programs | Cochrane et al. (2000)^98^   - To provide transitional support to individuals moving from inpatient settings to the community - To provide ongoing support to individuals with “difficult-to-control” behaviours, complex medication conditions, and other disabilities   Wasylenki et al. (2000)^101^   - The Seven Oaks Project employs resident-centered, psychosocial rehabilitation approaches, policies, and programming | Target program population:   - Individuals with complex, chronic conditions who require higher levels of staff support and program resources, as well as a structured and specialized environment | No outcomes were examined that are relevant to high support housing |
| Dorvil et al. (1997)^59^ | Family Housing – Caretaking Model:   - Foster homes with caregivers who are paid to house a restricted number of persons with mental illness; caregiving is considered a profit-making venture - Criticized for poor contacts between residents and home proprietors, poor social relationships between residents, regimentation and uniformity, lack of activity, and for lacking appropriate professional guidance   Family Housing – Professional Model:   - A professional is assigned to oversee client follow-up - Caregivers provide counselling, social protection, material services, recreation and cultural activities; social service staff are responsible for process control and support - Psychiatric emergency services are available 24/7 - Training is required for housing proprietors | Family Housing – Caretaking Model:   - The patient is to become part of their adopted family, and learn to “conduct himself in ways which will make him acceptable to those with whom he must live and, if feasible, work”   Family Housing – Professional Model:   - Developed to “correct the failures” of the caretaking model of family housing | Target program population:   - Patients with psychiatric disabilities | No outcomes were examined that are relevant to high support housing |
| Dorvil et al. (2005)^61^ | Custodial housing model:   - Long-term, “hospital-style” accommodation, in which services are offered by non-professionals - Model is described as offering “no responsibilities or learning” - Foster homes (or foster families, affiliated homes) and pavillons are provided as examples, and are described as being limited and extensive in their capacities, respectively | Although seen as an alternative to institutionalization, concerns regarding the perpetuation of institutionalization, contribution to problem behaviours among residents, and failure to provide positive supports and services have been raised | Target program population:   - People with mental illness | No outcomes were examined that are relevant to high support housing |
| Durbin et al. (2004)^51^ | *Homes for Special Care* (HSC):   - A type of board-and-care home, operated in association with provincial psychiatric hospitals - Onsite supervision provided by non-professionals; a hospital fieldworker is assigned to each resident to help with programs relating to adjustment and management - Meals, housekeeping, and laundry services provided - Psychiatric consultation available to clients - Level of care not consistently matched to level of need | - Established to provide a community-based residential care option for patients discharged from hospital; criticized for not promoting independence, resident choice and decision-making, and personal growth - Underlying assumption that target population “never improved or recovered, and required only custodial care” | Target program population:   - People with serious and persistent mental illness   Characteristics only provided for sample of HSC residents with a schizophrenia spectrum diagnosis (N=88):   - >65 years old: 26.1% - Gender: F=28.9% | - HSC residents experienced moderate impairment in psychiatric symptoms, had difficulties with self-care and meeting basic needs, had required supervision related to behaviour and medication management, and had few personal resources - Clinicians identified high levels of need among residents for medication monitoring and assessment/diagnosis and rehabilitation, which was mostly met; unmet needs included: psychotherapy/counselling, vocational and educational support - HSC was discussed as being “ill-equipped” to respond to the diverse needs of residents, and poorly positioned to provide intensive treatment and rehabilitation to those experiencing extreme difficulty; an emphasis on the need for greater integration and linkage with community resources, as well as trained onsite staff, was proposed |
| Edge & Wilton (2009)^71^ | Residential care facilities in Hamilton, Ontario:   - Previously referred to as privately owned lodging homes, residential care facilities provide food, accommodation, and rehabilitation services - Facilities have 8-40 beds or more, and facility operators are paid a per diem for each resident - Residents receive a monthly allowance for personal needs | Facilities were intended to be transitional, with residents moving on to more independent housing arrangements | Target program population:   - People with psychiatric disabilities, low-income elderly, people with physical and developmental disabilities   Characteristics provided for sample of residents (N=50):   - Mean age: 43 years - Gender: M=41, F=9 - Average length of occupancy: 4.5 years | - Housing concerns raised by residents included: lack of privacy, overcrowding, verbal harassment from staff and other residents, lack of safety and theft - 80% of residents were generally satisfied with their current housing overall - Whereas two-third of residents reported encouragement from facility staff to partake in programs outside of their housing, key informants noted that residents were often unable to apply learned skills - 60% of residents indicated a desire for more independent living; the rate was high among younger residents |
| Farrell & Aubry (2002)^42^ | *The Shared Responsibility for Care Program*:   - Pilot program developed by local mental health agencies, in partnership with community-based healthcare organizations and hospitals - Community-based healthcare organizations provide home-based nursing and support services - Designed to offer 24/7 onsite community-based supports | Not described | Target program population:   - People with severe and persistent mental illness   Characteristics provided for case study (N=1):   - Middle-aged adult with severe and persistent mental health problems - Frequently accessed psychiatric treatment - Long-standing difficulties with treatment compliance and in living independently | - The total costs of services (housing, social benefits, healthcare and social services, emergency services) while participating in the pilot program ($461/day) were comparable to community living ($459/day), but less than the costs of hospitalization ($522/day) - Higher housing costs were observed in the pre-participation period and were associated with emergency shelter stays and property damage; no such costs incurred when receiving services from the pilot program - Inpatient treatment and emergency services costs were lower when individuals were participating in the pilot program than the period of community living |
| Fleury et al. (2010)^124^  Piat et al. (2008)^112,113^ | Of the supervised housing program referenced in the article, three were considered high support housing (hostels, group homes, and foster homes), but these are not described | Not described | Target program population:   - People with serious mental illness   Fleury et al. (2010)^124^  Characteristics only provided for full sample of residents, which includes participants staying in other types of housing (N=315):   - Mean age: 49 years - Gender: M=57%, F=43% - Behavioural health: 75% had schizophrenia   Piat et al. (2008)^112^  Characteristics provided for residents of the three types of high support housing programs (N=198)   - Mean age: 52 years (hostel), 47 years (group homes), 51 years (foster home) - Gender: M=28, F=13 (hostel); M=28, F=24 (group home); M=48, F=57 (foster home) - Behavioural health: all participants had a diagnosis of serious mental illness (schizophrenia or schizoaffective disorder, bipolar disorder, or major depression); primary diagnosis of schizophrenia (75%)   Piat et al. (2008)^113^  Characteristics provided for residents of the three types of high support housing programs (N=315):   - Mean age: 51.7 years (pavillon), 47.3 years (group home), 50.8 (family-type resources) - Gender: M=68.3%, F=31.7% (pavillon); M=53.8%, F=46.2% (group homes); M=45.7%, F=54.3% (family-type resources) - Behavioural health: 75.4% had a diagnosis of schizophrenia | Fleury et al. (2010)^124^   - Housing type was not associated with adequacy of help   Piat et al. (2008)^112^   - Overall housing preferences among residents: independent apartments without subsidies (44%), social housing (18%), supervised apartments (15%), and foster homes (11%) - Overall housing preferences among case managers: supervised apartments (26%), foster homes (25%), group homes (15%), hostels (12%), and independent apartments without subsidies (11%) - 30% of residents already living in foster homes, and 48% of case managers, preferred this type of housing - 29% of residents already living in group homes, and 50% of case managers, indicated a preference for this type of housing - 29% of residents already living in hostels, and 67% of case managers, indicated a preference for this type of housing - Case managers preferred housing with greater structure and clinical supports compared to consumers   Piat et al. (2008)^113^   - Approximately 29.5% of respondents residing in family-type resources indicated a preference for this type of housing - Approximately 30% of respondents residing in group homes indicated a preference for this type of housing 29.3% of respondents residing in pavillons indicated a preference for this type of housing |
| Goering et al. (1992)^86,87^ | Goering et al. (1992)^86^   - Two congregate residential treatment programs are described (*Regeneration House Inc., Toronto East General and Orthopaedic Hospital Community Aftercare organization*) that offer permanent housing with three levels of support, including a 24-hour staffing level; residents may be transferred between homes or program levels as needs change - Residents are provided with opportunities to practice daily living skills, social and communication skills, decision-making and activities related to house management, and learn about and access community resources - Residents are paired a staff member or primary worker to work towards personal goals   Goering et al. (1992)^87^   - *Regeneration House*, a non-profit organization providing community housing to adults with histories of psychiatric illness, is described (Goering et al. 1992)^86^ - Operates five houses offering graduating levels of programming, support, and supervision - Residents participate in household chores and house meetings - No restrictions on length of stay; residents who benefit from the program can remain indefinitely | - To improve residents’ social, living, and coping skills, and to increase resident independence - To ensure that residents are well-linked to ongoing social support systems (family, friends, professionals, services) | Target program population:   - People with serious mental illness - Level I admission criteria: “10-year history of illness, five hospital admissions for psychiatric illness or inpatient treatment totalling at least one year” (Goering et al., 1992)^87^   Characteristics only provided for sample (N=42):   - Mean age: 34 years - Gender: Male=25, F=17 - Behavioural health: 30 had schizophrenia, 4 had a major affective disorder, 7 had other diagnoses - Mean length of stay: 17 months, with a maximum of 70 months | Goering et al. (1992)^86^   - Level I residents were found to have the greatest number of staff in their social networks and received the most amount of support, compared to residents in other housing with less support   Goering et al. (1992)^87^   - Focusing on program, staff perspectives, staff were aware of areas of what residents found attractive (e.g., social life, support of staff) and areas dissatisfaction (e.g., congregate/communal living, mandatory program activities) - Staff recognized improvements in residents’ life skills, insight and coping abilities, and the role of staff in facilitating change - Whereas residents felt that they had been helped by the program, staff were less optimistic about residents’ prospects |
| Heard et al. (2019)^104^ | - Transitional housing settings operated by St. Leonard’s Community Services, London and Region (SLCS) are described; SLCS is a community partner of the Southwest Centre for Forensic Mental Health Care - SLCS facilities offer secure, controlled, and highly structured residential settings with 24-hour staffing | - Transitional housing is described as typically offering time-limited tenure, with goals of enabling residents to move on to independent living - SLCS dedicated to promoting “positive change” in persons who are or may be in conflict with the law - Facilities follow an “accountability-driven approach” for transitioning to more independent housing in the community | Target program population:   - Individuals who have come in contact with the justice system in Ontario, Canada   Characteristics only provided for sample (N=6):   - Mean age: 47 years - Gender: M=3, F=3 - Mean length of stay at SLSC facilities: 1.8 years, with a low of 6 months to a high of 2.5 years - Mean tenure within the Forensic mental health system in Ontario: 7.6 years, with a range from 3.5 years to 11 years - Behavioural health: diagnoses included schizophrenia and schizoaffective disorder | - Residents described the importance of relationships with SLSC staff, valuing staff who were present and respectful - Sharing experiences with program staff and peers was described as improving quality of life among participants - Residents reported the value of being supported in their own personal growth and development, and described how residing at SLSC facilities enhanced self-belief, awareness, confidence, esteem, and hope - Participation in the program encouraged skills development, promoted self-confidence, and enhanced resiliency among residents and in their transition from a forensic mental health facility to more independent community tenure |
| Kidd et al. (2012)^96^ | *Centre for Addiction and Mental Health (CAMH) high-support housing initiative*:   - Initiative serving 18 clients with schizophrenia, with 18 units dispersed across two floors of a single building - 24/7 supports are provided by a community service organization - Case management, peer support, and psychiatric services are provided by the affiliated hospital, CAMH | - Aims to transition psychiatric inpatients out of hospital who may be better served within a community setting with appropriate supports - Draws on a recovery model with individualized goals and program planning | Target program population:   - Inpatients with severe mental illness residing in an Ontario mental health and addictions hospital   Characteristics of program (N=18):   - Age range: 39-58 years - Gender: M=11, F=7 - Mean length of stay in hospital: 5-25 years | - Staged move-in of residents was a successful strategy for relationship-building and facilitating community involvement - Per-diem costs of the high support housing initiative were significantly lower than inpatient hospitalization - Low rates of rehospitalization were found among high support housing residents discharged from hospital |
| Kirkpatrick & Byrne (2009, 2011)^88,89^ | *Housing with Outreach, Mobile and Engagement Services* (HOMES) Program:   - Program provides safe, secure, and affordable housing - Support services and staff are available 7 days a week, with 24-hour emergency services - Housing with supports is provided in three models, including single-room occupancy (i.e., individual rooms with private bathrooms, shared meals, and 24/7 onsite supports) - Provides access to physical health and psychiatric assessment and treatments - Following a Housing First philosophy, residents are not required to adhere to treatment prior to program entry | To advance the shift from shared accommodations in custodial lodging homes to supported housing | Target program population:   - Individuals with a history of homelessness and mental illness | Kirkpatrick & Byrne (2009)^88^   - Gary, a single room occupancy resident of the HOMES Program, was appreciative of the program, referencing the value or privacy and importance of social connection - The program provided Gary with employment opportunities and treatment   Kirkpatrick & Byrne (2011)^89^   - Having a place of one’s own was highly valued by residents, and was associated with privacy and a sense of control; for some, having one’s own place mean residents could reconnect with loved ones - Residents claimed that staff were respectful, “being there” for them 24/7 and were someone they could count on - Residents perceived the program to be empowering, enabling them in their “moving on”; the program provided residents with opportunities for skills development and growth (e.g., employing tenants as peer support workers) - Residents were grateful for and enjoyed the programs and events hosted by the program (e.g., karaoke), and noted the importance of social activities |
| Lariviere et al. (2002, 2006)^72,73^ | - Intermediate care facilities and private group homes (“pavillon”) are described, with nursing staff available 24/7; a staff:patient ratio of 1:5 to 1:15 is noted for intermediate care facilities, and a staff:patient ratio of 1:8 to 1:23 is noted for the pavillon setting - In both, supports for basic activities of daily living are adapted according to the resident’s level of functioning and needs - In both, rehabilitative services and activities are offered onsite; meals are provided - In both, residents are provided with opportunities to participate in instrumental activities of daily living (e.g., cleaning of common rooms) - In intermediate care facilities, psychiatrists act as consultants and meet with patients in-house   Lariviere et al. (2002)^72^   - Foster families are managed by private owners, with a maximum capacity of 9 individuals - Professional services are provided on a consultation basis | Not described | Target program population:   - Older adults with serious mental illness   Characteristics only provided for full sample (N=33):   - Mean age: 73.7 years - Gender: F=21, M=12 - Behavioural health: 22 had a psychiatric disorder, 7 had bipolar disorder, 1 had major depression, 3 had a personality disorder, 6 had dementia/organic brain syndrome, 8 had other diagnoses - Housing setting: 1 participant was transferred to a pavillon setting, 26 were transferred to intermediate care facilities, and none were transferred to live with foster families | Lariviere et al. (2002)^72^   - All facilities provided satisfactory living conditions and services, including day treatment, onsite rehabilitation, and recreation activities - Residents demonstrated stability in global, social, and activities of daily living functioning following discharge from psychiatric hospital; No significant differences were found in resident’s psychiatric/cognitive state over time - Residents required moderate assistance with activities of daily life (ADL) tasks, and occasional intervention for social problems - Residents were highly satisfied with their life in the community, and preferred living in their current accommodation than psychiatric hospital   Lariviere et al. (2006)^73^   - No significant deterioration in resident symptomatology, cognitive functioning, or activities ADL functioning observed, regardless of time spent in the community (6-12 months, 12-24 months, or ≥24 months) - Significant differences found between groups on social functioning, with deterioration seen among those living in the community for ≥24 months - No significant differences between groups in rates of rehospitalization; very few rehospitalizations were reported overall - No significant differences between groups in quality of life emerged; residents were highly satisfied with their residence and strongly preferred living in a community facility than psychiatric hospital |
| Lesage et al. (2003)^74^ | - Three types of supervised residential facilities (hostels, foster families, and group homes) are described - Hostels and foster families are privately operated, providing room and board and supervision with daily activities; professional staff visit weekly, and leisure, rehabilitative, and occupational activities may be provided | - Group homes have a rehabilitation model aimed at improving life skills and transitioning residents to less supervised settings - No details provided on hostels and foster families | Target program population:   - Adults with severe mental disorders | - Per 100,000 inhabitants, hostels were provided to 21 people, foster families to 40 people, and supervised group homes to 21 people - Per 100,000 inhabitants, 14 people estimated to need hostels, 26 need foster families, and 40 need supervised group homes |
| Lesage et al. (2008)^25^ | - Four tertiary psychiatric residential facilities in B.C. are described - Buildings each have ≤20 residents and provide private bedrooms, with shared bathrooms, kitchens, and common areas; multidisciplinary teams provide support and staffing ratio is “generally close to 1”; psychosocial rehabilitation programming is provided | Intensive treatment, psychosocial rehabilitation, and security needs are balanced with autonomy and a recovery orientation | Target Program Population:   - People with severe mental illness, including some of “the most difficult to treat patients” | - The mean cost per resident for one residential facility was CAD $350/day, which was similar to the per diem cost for the provincial psychiatric hospital in British Columbia |
| Lesage (2014)^115^ | Hostels, foster families, and supervised group homes listed as components of a balanced mental health system for people with severe mental illness, but not described | Not described | Target Program Population:   - People with severe mental illness | - Per 100,000 inhabitants, 14 hostels, 26 foster families, and 28 supervised group homes estimated to be needed - Estimated costs per person per year were $49,793 for supervised group homes, $35,616 for hostels, and $7,746 for foster families |
| Nelson et al. (1992, 1997, 1999)^52-54^ | Board-and-care homes (BCH)   - Programs provide around-the-clock staffing, and are privately owned and operated for-profit; intended to be permanent housing - Houses up to 30 occupants; meals and some recreation provided - Residents are offered opportunities to participate in weekly social and recreational activities - Criticized for being “noisy,” “occasionally run-down,” “crowded,” “less physically comfortable” | Do not have explicit rehabilitation goals or methods, but instead aim to provide comfort, support, and meals to residents | Target program population:   - Individuals with long-term and serious mental health problems   Nelson et al. (1992)^52^  Characteristics only provided for full sample (N=66):   - Mean age: 34.4 years - Gender: M=40, F=26 - Behavioural health: “diagnosed as having schizophrenia, chronic depression, or manic-depression”   Nelson et al. (1997)^53^  Characteristics only provided for sample of BCH residents (N=25):   - Mean age: 45.0 years - Gender: M=11, F=14 - Mean length of stay: 39.1 months   Nelson et al. (1999)^54^  Characteristics only provided for sample of BCH residents (N=31):   - Mean age: 44.7 years - Gender: F=22 | Nelson et al. (1992)^52^   - Board-and-care home (BCH) residents had fewer family members, friends, and professionals in their networks, but these findings were not significantly different than residents of group homes (GH) and supportive apartments (SA) - BCH residents received support from fewer friends and professionals than GH and SA residents - BCH residents provided and less frequently received support, and reported lower levels of community integration and independent functioning compared to GH and SA residents   Nelson et al. (1997)^53^   - SA and GH residents were more likely to have their own room, more control in decision-making, and fewer restrictive housing rules than those in BCH - No differences were found between groups on housing concerns, personal empowerment, community integration, and quality of life - BCH residents reported more staff and social support, as well as less emotional abuse, than SA residents - Fewer BCH residents reported having interpersonal conflict and relationship problems with their roommates/living companions compared to SA residents - GH and SA residents had greater improvements in staff-rated independent functioning than BCH residents   Nelson et al. (1999)^54^   - BCH facilities had more residents than GH and SA buildings - BCH residents were found to have lower levels of housing control and be less likely to have their own rooms compared to GH and SA residents - BCH residents reported lower levels of personal empowerment and higher levels of emotional well-being compared to GH and SA residents - Social support was similar between BCH, GH, and SA residents |
| Nelson et al. (2003)^55^ | *Homes for Special Care* (HSC):   - Large, single-site houses operated for-profit - Houses 4-20 people in either private or shared bedrooms - Accommodation, meals, and 24-hour staffing provided | Not described | Target program population:   - Psychiatric consumer/survivors   Characteristics provided for sample of HSC residents (N=91):   - Mean age: 43.1 years - Gender: M=38, F=53 - Behavioural health: 51 had schizophrenia, 15 had a mood disorder, 20 had another mental illness | - Most residents preferred to live in their own apartment or house, whereas 10% preferred living in HSC - Almost half of residents wanted to live alone, whereas 23% preferred to live with other people who had mental illness - Privacy, freedom, independence, and ownership were important qualities in preferred living situations - 70% of participants reported around-the-clock staffing to be an important support - Rates of housing satisfaction among HSC residents were greater than those living in shelters |
| Nelson (2010)^75^ | Custodial housing (i.e., board-and-care homes, foster families):   - The consumer is a “patient” and staff is a “care provider” - Staff maintain control over housing, and provide in-house care services; medications and meals are typically provided - Criticisms surrounding privacy, the physical quality of housing, and a lack of resident control have been noted | Described as offering minimal rehabilitation and supports to foster resident independence and community integration | Target program population:   - People with serious mental illness | Findings were previously reported in Nelson et al. (1997, 2003)^53,55^, Piat et al. (2008)^67^, and Trainor et al. (1993)^77^ |
| Newton & Schieldrop (2005)^100^ | Seven Oaks, Phase 1:   - Pilot project from 1994-2002 designed as transitional accommodation, with 12 beds - Each resident had their own bedroom; residents were responsible for their personal hygiene and cleanliness of their room, and had a budget for food and household supplies - Staff, including a nurse and healthcare worker, were available 24/7; supervisors, a social worker, an activity coordinator, and psychiatrist were also available - Each residence had a “resident council” that was supervised by staff and gave residents a degree of control over facility operations   Seven Oaks, Phase 2:   - Expanded in 2002 to include a 38-bed, 5-unit facility built on the site of the 12-bed residence - Residents have their own bedroom with bathroom, and share a living/dining area, kitchen, and laundry - Various activities, programming, and educational opportunities are available to residents, including meal planning/preparation and occupational programming - Facility is staffed by nurses and psychiatric rehabilitation works, as well as psychiatrists, a social worker, and an occupational therapist | Seven Oaks, Phase 1:   - Initial function was to provide care and treatment to “the most difficult patients” - Emphasized individual care planning   Seven Oaks, Phase 2:   - Emphasized rehabilitation | Target program population:   - People with serious and persistent mental illness who require 24/7 support   Seven Oaks, Phase 1 (N=38):   - Age range: 20-65 years - Behavioural health: >90% had “a primary diagnosis of schizophrenia with social-emotional disorders” | - Preliminary results from a study of Seven Oaks (Phase 2) indicated positive outcomes for patients, including successful transfer of patients from hospital to Seven Oaks, regular transfer of patients from Seven Oaks to community settings, more opportunities to develop daily living skills compared to hospital, and greater overall patient satisfaction - The cost of the Seven Oaks program was comparable to hospital, with the per diem for 38 beds equalling $337/day |
| Patterson et al. (2013, 2014)^78,79^  Rezansoff et al. (2017)^80^  Russolillo et al. (2014)^81^  Somers et al. (2013, 2015, 2017)^82-84^ | Congregate Housing First (HF):   - Single-site housing project with 24/7 onsite supports, located in a building with the capacity to house ≥100 occupants - Multidisciplinary teams consist of health care professionals, peer support workers, and case managers, with a client-staff ratio of 12:1; supports and services are comparable to Assertive Community Treatment - Continuous staff presence at the front desk/reception area - Building facilities, including a central kitchen and meal area, medication room, and recreational areas, are designed to support residents and promote positive community culture - Residents are provided opportunities to engage in part-time work within the building and/or in the community - Medical examination room and pharmacy available onsite - Tenancy is not contingent upon compliance with therapeutic objectives | - Services modeled on the approach developed by Pathways to Housing, including an emphasis on promoting individual choice and adoption of a harm reduction orientation and practices in relation to addiction - To provide barrier-free access to accommodation and recovery-oriented supports | Target program population:   - People experiencing homelessness and mental illness   Patterson et al. (2013)^78^  Characteristics only provided for sample of “High Needs” group (N=297):   - Mean age: 39.7 years - Gender: M=218 - Mean lifetime duration of homelessness: 62 months - Mental disorders (“severe cluster,” including “at least one of psychotic disorder, mood disorder with psychotic features, and manic/hypomanic episode”): 272 - Housing setting: 107 participants assigned to CONG HF   Patterson et al. (2014)^79^  Characteristics only provided for full sample, including “High Needs” and “Moderate Needs” groups (N=497):   - Mean age: 40.8 years - Gender: M=73% - Mental disorders (“severe cluster”): 73% - Housing setting: 107 participants assigned to CONG HF   Rezansoff et al. (2017)^80^  Characteristics only provided for subsample of CONG HF who initiated antipsychotic pharmacotherapy prior to recruitment (N=57):   - Mean age at recruitment: 38.7 years - Gender: F=14 - Lifetime duration of homelessness (>3 years): 21 - Behavioural health: all had a diagnosis of schizophrenia   Russolillo et al. (2014)^81^  Characteristics only provided for sample of residents who provided consent and had valid health information numbers (N=223):   - Mean age: 40 years - Gender: Male=74% - Behavioural health: 92% had at least one of current psychotic disorder, mood disorder with psychotic features, or hypomanic or manic episode - Housing: 89 participants assigned to CONG HF   Somers et al. (2013)^82^  Characteristics only provided for sample of CONG HF residents with least one recorded prior contact in the Provincial justice system (N=78):   - Mean age at enrolment: 39.8 years - Gender: M=62 - Lifetime duration of homelessness: 60.8 months - Mental disorders (“severe cluster”): 70   Somers et al. (2015, 2017)^83,84^  Characteristics only provided for sample of CONG HF (N=107):   - Mean age: 40 years - Gender: M=82 - Lifetime duration of homelessness: 36 months - Diagnosis: 79 had a psychotic disorder, 35 had a major depressive episode, 25 had a manic or hypomanic episode, 20 had a mood disorder with psychotic features, 67 had substance dependence, and 28 had alcohol dependence | Patterson et al. (2013)^78^   - Congregate HF residents reported significantly greater overall quality of life, personal safety, and living situation satisfaction compared to treatment as usual (TAU) at 6 and 12 months   Patterson et al. (2014)^79^   - No significant differences were found in physical or psychological community integration between congregate HF and all other arms over time - Congregate HF residents were more likely to report knowing their neighbours, but not interacting with them or the emotional components of community compared to participants in the high needs TAU group   Rezansoff et al. (2017)^80^   - Medication adherence did not significantly differ between congregate HF and TAU - Scattered-site HF had highest number of antipsychotic-related pharmacy transactions (180 per person-year), followed by congregate HF (167 per person-year) and TAU (99 per person-year)   Russolillo et al. (2014)^81^   - Congregate HF was associated with marginally lower emergency department use in the years before and after study enrolment when compared to TAU, but this was not statistically significant   Somers et al. (2013)^82^   - Congregate HF was associated with marginally significant reductions in convicted sentences compared to TAU - Congregate HF residents had a mean 0.55 reconvictions following randomization compared to scattered-site HF (0.29)   Somers et al. (2015)^83^   - No significant differences were found in the effectiveness of congregate HF compared to TAU on daily substance use after 12 and 24 months - Congregate HF residents spent more time in stable housing (74.3%) than TAU (27.9%) after 24 months; housing stability between congregate HF and scattered-site HF was similar   Somers et al. (2017)^84^   - Congregate HF residents had significant improvements in severity of disability, psychological community integration, and recovery from baseline to 24 months compared to TAU; congregate HF residents spent more time in stable housing TAU after 24 months - No differences were found in the effectiveness of congregate HF compared to TAU on physical community integration, psychiatric symptoms, overall health, food security, substance use problems, or quality of life |
| Petersen et al. (2013)^19^ | - Tertiary Psychiatric Residential Facilities provide 24-hour multidisciplinary staffing, with staffing ratios comparable to acute care wards - Facilities typically have less than 25-beds, and are operated by local health authorities as smaller, more “home-like” alternatives to hospitals - Facilities incorporate programs focused on clinical and rehabilitation care | Aim to provide intensive long-term treatment and rehabilitation | Target program population:   - Patients with severe and persistent mental disorders who cannot be supported in lower intensity residential facilities or housing alternatives   Characteristics only provided for sample of participants (N=189):   - Mean age: 47.2 years - Gender: M=114, F=75 - Behavioural health: 151 had a schizophrenia spectrum disorder | - Over a 5-year period, many residents gradually transitioned to facilities with lower levels of support and onsite programming, though 70.7% of residents remained in facilities with 24-hour staffing at the fifth follow-up point - The percentage of residents residing in Tertiary Psychiatric Residential Facilities decreased over time |
| Piat et al. (2002)^62^ | - Foster home that included a caretaker who lived on the premises and provided room and board, and supervision to residents - The caregiver was on-call for residents 24/7; extended family of the caregiver also provided support in the home - The caregiver supported implementation of residents’ treatment plans and received supervision from a housing worker - A nursing assistant from a large teaching hospital was also onsite full-time during typical work hours - Residents were followed by a hospital-based multidisciplinary team that visited biweekly | Not described, though confusion about mandate (resident rehabilitation versus maintenance) was an evaluation finding | Target program population:   - People with a severe and persistent mental illness who had been hospitalized, with a history of past challenges maintaining housing   Characteristics of the 6 residents:   - Age range: 24-54 years - Gender: M=5, F=1 - All had diagnoses of schizophrenia - All had repeated hospitalizations and past housing difficulties | - Residents had fewer days in hospital, but more emergency department visits after moving into the foster home (estimated hospital service use was $455,000 for the year pre-placement, compared to $86,800 for the year post-placement) - Residents expressed housing satisfaction due to the home-like environment, a sense of belonging, and appreciation of the professional support; sources of dissatisfaction were the: building’s inconvenient location, group-living environment, noisiness, food quality, and smoking policies - Five of the six residents remained at the foster home after 24 months |
| Piat et al. (2007, 2008, 2009)^66-69^ | - Foster homes are the oldest form of housing for persons with serious mental illness; also known as “family type residences” - Foster homes are operated by non-professional caregivers and are a public sector service regulated by law in Quebec - Foster homes can accommodate up to 9 residents; caregivers are paid a per-diem for supporting people in their homes - Foster home caregivers are recruited by mental health workers; consumers are matched and placed by mental health workers - A case manager is assigned to each resident; social workers and caregivers work collaboratively to develop service plans for each resident - Caregivers (or a replacement) must be available (on-call) or physically present 24/7 | - Foster homes have been described as evolving from a “caretaking” model to a “professional” model, with professionals (social workers, nurses) supervising over and linking foster homes with formal services - Foster homes caregivers mandated to reintegrate residents into the community, though have been previously criticized for being “too structured and controlling” | Target program population:   - People with serious mental illness   Piat et al. (2007)^66^  Not described  Piat et al. (2008)^67^  Characteristics only provided for sample of foster home residents (N=33):   - Mean age: 52 years - Gender: M=20, F=13 - Behavioural health: 14 had schizophrenia, and 13 had a mood disorder   Piat et al. (2008)^68^  Not described  Piat et al. (2009)^69^  Characteristics only provided for sample of foster home residents (N=102):   - Mean age: 54 years - Gender: M=45%, F=55% - Mean length of occupancy: 5.4 years | Piat et al. (2007)^66^   - Caregivers described the qualities required to help people with mental illness; 21 categories and themes were identified - Values and qualities provide caregivers with a “professional” or “vocational” orientation   Piat et al. (2008)^67^   - Residents described needs for security, stability, and support, which could conflict with a desire for greater autonomy, dilemmas with communal living (e.g., cultural differences, negative behaviours, interpersonal difficulties), and the struggle to envisage a positive future - Foster homes provided opportunities for social interaction, friendship, and mutual support according to residents   Piat et al. (2008)^68^   - Caregivers described their jobs as professional work and viewed themselves as “dedicated helpers” who were “the real frontline workers,” referencing the considerable responsibility, time, and energy that is required to support for foster home residents - Caregivers claimed to be available 24/7, provide more personalized care, and know residents better than do social workers; highlighted that sharing their homes and family life with residents enabled positive, accessible, and affectionate relationships   Piat et al. (2009)^69^   - 89.0% of foster home residents preferred living in a foster home compared to hospital, though 59% did not choose to live in such a residence; almost all residents (95%) recommended foster homes for housing - >50% of residents reported adapting to foster home living, whereas 26.7% reported adaptation difficulties, including with other residents (9%), and the caregiver or home environment (6%) - Residents expressed dissatisfaction with the rules and structure (19%), and presence of other residents (11%) - Caregivers reported high levels of autonomy in the majority of homes, and aspects of life within the homes; foster homes were perceived to provide greater autonomy than hospitals, hostels, and group homes |
| Piat et al. (2004)^63^ | - Foster homes are the primary type of housing for most patients discharged from psychiatric hospitals in Montreal, Canada and are regulated by law - Operated by non-professionals, though support groups and training are offered to foster home caregivers - Caregivers work in collaboration with multi-disciplinary teams (social workers, resource workers), who oversee the well-being of foster home residents - Foster homes can accommodate up to 9 residents; caregivers receive a per-diem for each resident living in their home - A centralized hospital-based committee oversees transitions to foster homes | Foster homes have been described as evolving from a “caretaking” model to a “professional” model, with health professionals supervising and connecting foster homes to formal services | Target program population:   - People with serious mental illness who may be unable to maintain housing independently in the community   Characteristics only provided for sample of foster home residents (N=25):   - Age range: 18-64 years - Gender: M=14, F=11 - All had a diagnosis of a serious mental illness (i.e., schizophrenia, bipolar disorders, major depression) | - Location, cleanliness, maintenance, and lack of private space were identified issues with foster home physical environments - Residents and professionals reported issues around the organization of the foster home, including rules and the accessibility of the home; residents cited issues of autonomy - Family members expressed a desire for closer monitoring and supervision of their relatives living in the foster home - Professionals and family members indicated a need for caregivers to have more knowledge and training on mental health - Residents and caregivers emphasized the importance of integration and relationships within the foster home; professionals stressed the importance of integrating residents into the broader community - Caregivers viewed themselves as “both the parents and rehabilitation agents” of their foster homes |
| Piat et al. (2005)^64^ | - Foster homes are private homes that are managed by non-professionals and are governed by law in Quebec - Foster homes can accommodate up to 9 residents - Managers of foster homes are described as having a role in integrating people with mental illness into the community | - Aims to provide a safe and secure living environment and promote community integration - Foster homes have been described as moving from a traditional “caretaking” model to a “rehabilitation” model, where health professionals are considered as intermediaries linking residence managers and formal services | Target program population:   - People with serious mental disorders | - Foster home managers discussed the positive impacts of transformations in the health and social services networks in relation to their roles and responsibilities (e.g., feeling of doing more rewarding work), and on resident outcomes (e.g., improved quality of life) - Foster home residents experienced a “more normal” existence when leaving hospital, according to managers; foster home residents progressed more quickly outside of institutions (e.g., learning to clean and cook), according to managers - Managers indicated the negative impacts of transformations in the health and social services networks, including increased workload, insufficient resources and services, and taking on “heavier” or more complex patients, among others; a shortage of professional staff (i.e., doctors, nurses) and crisis services were noted, as well as supports for managers (e.g., respite) |
| Piat et al. (2005)^65^ | - Foster homes are family-type resources that can accommodate up to 9 people; residents generally stay in foster homes for 3-5 years - Foster home managers, who are mostly women, supervise these homes; managers do not have recognized training - Professional monitoring is provided by multi-disciplinary teams, including social workers, nurses, occupational therapists, and psychiatrists; social workers are the main link between foster home managers and clinical teams | Not described | Target program population:   - People with serious mental disorders | - Interviews with social workers generally revealed shortcomings and frustrations in the partnership between foster home managers and social workers; for example, managers may adopt a “controlling” approach to social workers, and that communication may be difficult between parties - Overall, respondents noted that foster home managers receive little support from various stakeholders, and may be considered solely responsible for their residents; respondents were concerned of the working conditions of managers - The majority of respondents suggested that managers ought to be recognized as full, valued members of the clinical team |
| Piat et al. (2011)^43^ | - Housing was organized by level of supervision, including a high support housing category: short-term transitional housing with intensive supervision (high supervision) - Mental health professionals from affiliated hospitals supervise the residences - Different housing categories are operated by either 24/7 staff, nonprofessional caregivers, or off-site staff - Residents are referred by the hospital system and followed by multidisciplinary teams | Not described | Target program population:   - People with serious mental illness   Characteristics only provided for full sample (N=40):   - Mean age: 46 years - Gender: M=57.5% - Behavioural health: 60% had schizophrenia, 15% had major depression, 10% had a bipolar disorder - Housing history: participants lived between 1-7 years in current housing, with an average tenure just under 4 years | - 78% of residents recommended their current housing as a “good place to live,” but only 40% wanted to make their living situation permanent - Residents of highly supervised housing primarily identified family members (n=8), professionals (n=4), residence caregivers (n=3), and themselves (n=2) as the people who most believed in them and their recovery; no resident selected friends as the people who believed in them and their recovery |
| Piat et al. (2015)^70^ | - Congregate housing includes a network of not-for-profit foster homes and group homes - Services are tied to congregate housing, with non-professional caregivers providing 24/7 accommodation and psychosocial rehabilitation services - Housing proprietors are recruited and supervised by hospital professionals; residents are followed by multi-disciplinary hospital teams | Not described | Target program population:   - People with serious mental illness, who may be unable to maintain themselves independently in the community   Characteristics of housing residents (N=188):   - Mean age: 49.5 years - Gender: M=123, F=63 - Mean length of occupancy: 7 years - All had a diagnosis of serious mental illness (schizophrenia, bipolar disorder, or major depression) | - Residents rated the recovery orientation of their homes significantly lower than did proprietors - Proprietors felt that residents need to be more involved in staff training and education programs; proprietors expressed concerns about their own lack of training on cultural competency - Residents reported a lack of support in exploring alternative housing options, self-help connections, and peer support resources - There was agreement in resident and proprietor perspectives that residents have little choice in case management or treatment planning, and beliefs among proprietors that residents are unable to manage their symptoms |
| Rudoler et al. (2018)^97^ | *Alternative Level of Care (ALC) High Support Housing Initiative*:   - Housing component includes 24/7 access to trained, onsite staff; medication management and monitoring; personal support workers; peer support; meals; flexible and individualized spaces; substance use, stress, and anger management supports - The initiative also includes a flexible financial resource fund available for housing providers to develop and implement individualized care plans | - Aims to transition ALC inpatients residing in hospital into the community, and to prepare them for more independent living - Uses a recovery-oriented approach | Target program population:   - ALC psychiatric hospital patients   Characteristics only provided for sample (i.e., clients with ≥90 day lengths of stay in hospital, who were discharged to assisted living, board-and-care homes, mental health residences, group homes for persons with physical disabilities, or settings for persons with intellectual disabilities (N=69)):   - Age at assessment: 46.7 years - Gender: F=24.6% - ALC hospital stay: 238.8 days - Length of hospital stay: 692.3 days   Data not provided for only participants who were discharged to high support housing programs | - The high support housing initiative resulted in greater cost savings relative to inpatient care, with an estimated average cost saving of $140-$160 (approximately $51,000-$58,000 annually, per client) |
| Trainor et al. (1993)^77^ | Custodial housing models:   - Considered to be a primary housing option for former psychiatric patients in Canada - Tend to be large facilities often operated and staffed by non-professionals, and offer minimal programming, interpersonal contacts, and a regimen of daily activities - Economic factors have been cited as discouraging operators from providing rehabilitative services | Custodial models are not typically focused on rehabilitation and concerns have been raised that the models replicate the characteristics of larger psychiatric institutions | Target program population:   - People with mental illness | - Past research on custodial housing indicates that residents tend to be isolated and lonely, inactive in both the facility and local community, and dissatisfied in their housing situation |
| Vandevooren et al. (2007)^107^ | *Steele Street Residential Treatment and Rehabilitation Program:*   - Developed in response to the downsizing of a local psychiatric hospital - Offers a range of services, including treatment, rehabilitation, and supports, which are provided by multidisciplinary teams - Program houses up to 9 individuals | To prepare residents for more independent living | Target program population:   - Individuals with highly complex needs   Characteristics only provided for sample of residents who had completed the program (N=25):   - Mean age: 39 years - Mean length of stay in program: 13.9 months - Behavioural health: 67% had schizophrenia, 73% had a secondary diagnosis | - Participation in the program resulted in significant improvements in community tenure and individuals’ ability to live in more independent settings, reduced hospital admissions and symptoms, and improved improvements in functioning |
| Yamin et al. (2014)^111^ | *Peer Support Housing (PSH):*   - Structured, single-site housing with 6 large apartments; one apartment is occupied by a “peer support couple” who also serve as building superintendents - Building is owned by a private investor and leases were held by the At Home/Chez Soi program - Apartments are self-contained units, each with a kitchen, a bathroom, a living room, and one bedroom - Peer support is offered onsite by the peer support couple, as well as home visits from an Assertive Community Treatment team - PSH is used as emergency housing for individuals waiting for available apartments - Residents who achieve housing stability at the PSH can remain indefinitely, should program staff believe that they could not sustain stability in scattered-site apartments | - PSH services modeled on the Pathways to Housing approach - The goal of PSH is to house individuals who have experienced continued housing instability in HF | Target program population:   - PHS exclusively houses HF participants who had experienced multiple evictions, and had not secured stable housing over the course of one year of involvement with the HF program | - Current and former residents described the Peer Supportive Housing model as “as place to stay” (temporarily or permanently) with supports, and some described it as “last resort housing” for those who have been frequently evicted - Perceived strengths were identified, and include: the physical environment, with residents viewing Peer Supportive Housing as “calm, small, and convenient as all amenities were on site”; the additional structure and supervision provided, with residents grateful for the safety features in the building; on-site services and supports, including positive relationships among residents - Perceived weaknesses were identified, and include: aggressive behaviours exhibited by residents and problematic visitors; overly restrictive rules, according to residents; temporary residency, despite wanting to stay permanently at the Peer Supportive Housing; the location of the building, with residents describing it as “not ideal” - Program staff and residents alike believed that the Peer Supportive Housing model helped residents achieve stability; residents highlighted stability in financial, medical, and psychological aspects of life, and program staff attributed this to the structure of and support received in Peer Supportive Housing |

**Supplemental Table 8.** Description of High Support Housing Program and Outcomes (Grey Literature)

| **Authors (Year)** | **Description of Housing Model/Program** | **Objectives/Values of Housing Program** | **Target Program Population & Resident Characteristics** | **Key Findings** |
| --- | --- | --- | --- | --- |
| Addictions and Mental Health Ontario et al. (2018)^108^ | *Strachan House:*   - 3-level, single-site housing operated by the Homes First Society; 83 units reserved for long-term tenants and 5 emergency shelter beds - Building is divided up into smaller “neighbourhoods,” consisting of 5-8 private units and shared spaces (kitchen, washroom) - 24/7 onsite support is provided by community support worker; support is also provided by hoarding and housekeeping specialists; onsite medical support provided by partner service providers - Life skills programs are provided to assist tenants with daily activities of daily living, improve financial literacy, and teachings on tenant obligations | - To create “a community within a community in a changing neighbourhood” - Practices “assertive tolerance” principles to accommodate and support individuals with complex needs; features of this include: following a harm reduction approach, using staff meetings to debrief around troubling behaviour, and using landlord function as a strategy to engage with tenants - Considered to be “no barrier housing,” in that tenants are able to choose how much or how little support they want to receive | Target program population:   - People who are chronically homelessness and are considered “hardest to house,” with severe mental health and addiction issues - Population has difficulty securing and maintaining housing | - 96% of Homes First Society – Strachan House residents remained stably housed in 2016 |
| Alberta Health Services (2014)^125^ | A conceptual model of housing and supports for home living, supportive living, and facility living is described, and includes:  Supportive Living levels 1-4D:   - Includes Residential Living, Lodge Level Living, Assisted Living, and Enhanced Assisted Living - 24/7 availability of staff in Residential Living and Lodge Level Living; meal services are provided - Can include on-site recovery programming (Residential Living), individualized recovery planning (Lodge Level Living), and in-reached recovery programming/services (Assisted Living, Enhanced Assisted Living)   Community Residential Treatment:   - Facility-based, providing individualized and multi-disciplinary care - Rehabilitation programming promote independent living - Full meal service, housekeeping, laundry and linen services are provided - 24/7 availability of professional staff and security services | Supportive Living levels 1-4D:   - Emphasis on providing an array of housing options and supports that are least restrictive and promote recovery - Based on the premise that clients should be able to choose an option that “best-fit” their diagnosis and support needs   Community Residential Treatment:   - Not described | Target program population:  Supportive Living levels 1-4D:   - Individuals requiring more support to live in the community - Target populations vary by level   Community Residential Treatment:   - Individuals with severe and persistent mental illness and/or addictions, among others | No outcomes were examined that are relevant to high support housing |
| Butterill et al. (2009)^33^ | - High support housing is conceptualized as integrating housing and treatment components in secure, “home-like” settings - High support housing provides 24/7 supervision and monitoring - Two Transitional Rehabilitation Housing Programs (TRHP) are described, with an intended length of stay of 12 months - One TRHP unit maintains a staff ratio of 1:5 (Toronto), and the other offers both congregate (four-bedroom house) and scattered living arrangements (Ottawa); both programs partner with mental health agencies and hospitals to deliver services | - To transition individuals on to more independent living - To support individuals in their transfer to “generic housing”; recovery and psychosocial rehabilitation are considered the “cornerstones of intervention” (TRHP) | Target program population:   - ALC/Long-stay hospital patients - Persons in forensic hospital units, who are allowed community placement under the Ontario Review Board (TRHP) | - A lack/inadequate supply of high support housing with 24-hour staffing was highlighted - Care providers, policy-makers, and other stakeholders suggested that high support housing programming should: be client-centered and recovery-orients; follow a holistic, biopsychosocial approach; be linked with primary care, among other features - An ideal model of high support housing was proposed based on participant feedback, and included: 24/7 supervision and monitoring; multidisciplinary, highly trained staff; a “home-like” environment that offers safety and security for staff, residents, and community; resident privacy, as well as shared areas - Average cost per diem of high support housing was found to less ($102.70) than both an inpatient bed in an acute care hospital ($1048) and a bed in a tertiary care hospital ($665) |
| Calgary Homeless Foundation (2021)^85^ | High support housing programs are described, and include:  *Aurora on the Park and Francis Manor – Alpha House*:   - Buildings consisting of self-contained, bachelors’ or one-bedroom units - Residents have access to volunteer, employment, and recreational programming, as well we Indigenous Cultural Reconnection Coordinators and peer support workers; meals are provided - 24-hour support available; nurse (weekly) and doctor (bi-weekly) visits are provided onsite, through partnerships with local organizations - Money, alcohol, and tobacco management supports available; harm reduction supplies onsite - No limit on length of stay   *The Prelude and Abbeydale Place – The Alex*   - Buildings consisting of 20+ units, offering 24/7 staffing, secure and controlled entry, and supports; meals provided - Case management, recreation, and family supports provided - Medication and psychiatric supports offered through medical clinic - Harm reduction practices used on a case-by-case basis, and each resident works closely with a case manager to plan for recovery and self-sufficiency - No limit on length of stay   *Stepping Stone Manor – CASS:*   - Long-term housing and supports dedicated to 28 adults - Support services are readily available to residents and offered 24-hours; housing is not contingent on participation in services - Activities and group work offered residents, depending on their readiness and availability; staff primarily provide coaching and mentoring support - “Eviction prevention” programming focused on nutrition and basic housekeeping - No limit on length of stay | All programs deliver place-based supportive housing, or case management and housing supports, with goals to achieve housing stability and independence  Aurora on the Park and Francis Manor – Alpha House:   - Focused on principles of harm reduction (Aurora on the Park) and helping individuals achieve housing stability (Francis Manor)   The Prelude and Abbeydale Place – The Alex   - Adheres to principles of harm reduction, intensive case management, and Housing First   Stepping Stone Manor – CASS:   - Case management is based on participant-directed goal setting and adopts a harm reduction approach | Target program population:  Aurora on the Park and Francis Manor – Alpha House:   - Individuals who have experienced chronic homelessness, and who have not succeeded with scattered-site Housing First programs   The Prelude and Abbeydale Place – The Alex   - Individuals experiencing a range of issues, including mental health and substance use disorders, trauma, long-term effects of homelessness, as well as medical challenges (The Prelude) - Individuals who, historically, have not been able to maintain housing in scattered site programs (Abbeydale Place)   Stepping Stone Manor – CASS:   - Individuals experiencing homelessness and major disabling conditions | No outcomes were examined that are relevant to high support housing |
| Centre for Addiction and Mental Health (2002)^47^ | Custodial Models, including *Homes for Special Care,* are described:   - Originally intended to support bed closures, and understood as a combination of models; regulated, but resistant to change - Primarily custodial, with limited rehabilitation focus; a common basket of services are provided to all residents - Operators must provide consistent services to all residents, emphasizing standards for meals, supervision, and laundry | Program discourages rehabilitation initiatives | Target program population:   - People with mental illness | - At the time of report, custodial housing accounted for over 4,800 units, or about 44% of total dedicated beds |
| Centre for Addiction and Mental Health (2005)^91^ | Several programs are described, including *COTA Comprehensive Rehabilitation and Mental Health Services – Adam’s House; LOFT Community Services: Wilkinson Housing and Support Services – Beaty House; Pilot Place*:   - All programs offer supportive housing for individuals with mental health concerns; programs offer 24/7, onsite support - Bed capacities range from 7 to 50 beds, with programs offering shared and single rooms - Many programs provide meal services; *Pilot Place* involves residents in meal preparation - While residents generally manage their own medication, staff assist if needed; recreational programming, personal care, substance use counselling, employment services, and cultural sensitivity training offered in select programs - Services like case management, general counseling, respite care, crisis intervention, and life-skills training mentioned in select programs | Several programs described to follow a harm reduction philosophy (COTA Comprehensive Rehabilitation and Mental Health Services - Adam’s House; LOFT Community Services: Wilkinson Housing and Support Services—Beaty House; Regeneration House Inc.: Homelessness Initiatives Program; Pilot Place) | Target program population:   - Varies by program, and includes: adults (18+ years) with mental health concerns, including a focus on schizophrenia; and older adults (50+ years) with specific care needs | No outcomes were examined that are relevant to high support housing |
| Centre for Addiction and Mental Health (2010)^95^ | *CAMH Schizophrenia, High Support Housing Initiative*:   - The Centre for Addiction and Mental Health (CAMH) partnered with Homes First and Pilot Place to offer high support housing services to long-stay ALC clients in the CAMH Schizophrenia Program | Not described | Target program population:   - ALC clients   Study Sample (N=9):  Residents discharged from CAMH Integrated Rehabilitation Unit, who had an average length of stay of ~9 years | - Reporting on a sample of the first 9 residents of 90 Shuter St., the initial year of operation was considered “highly successful” - All residents successfully transitioned out of hospital, and made significant gains in their sense of wellness and community engagement; no hospital readmissions were reported within the first year - Cost of services at 90 Shuter St. were significantly less than maintaining in-patient level of support (~$76/day, compared to $698/day for a tertiary bed); the per diem cost at 90 Shuter St. considered substantially less than the provincial mean cost of high support housing (~$100/day) - Program residents were happy with the quality of the physical space, the food and services provided, as well as amenities available; residents got along well with peers and valued relationships with program staff - Residents expressed a desire for greater autonomy (e.g., to be involved with employment) and control over finances; some had goals towards having their own apartments with less support - Residents reported making progress in their recovery (e.g., greater social and community involvement, improved psychological wellness), with no resident reporting a decline in any area; staff and mangers recognized similar gains |
| Centre for Addiction and Mental Health (2012)^109^ | - High support model of housing provides recovery-oriented services and support to complex clients; speciality trained, onsite staff - Short-term to long-term stay; housing in a clustered setting, with 24-hour staff availability - Four housing sites are identified but not described, though all partner with CAMH and other agencies: the *Transitional Rehabilitation Housing Program, the Stepping Stone Project, a housing program at 90 Shuter Street*, and a housing program at *1011 Lansdowne* | Not described | Target Program Population:   - People with mental illness | - Waitlist exceeds 300 for those in need of high-support housing in Toronto - Residents discussed the value of having secure housing, perceiving this as opportunity to focus on physical and mental health recovery; most residents stated that their health and quality of life improved since moving into their accommodations - Staff believed that secure housing provided clients with “an opportunity to rebuild” one’s health and social relationships; residents and clients viewed their current housing situation as part of a transition towards fully independent living - Residents noted the importance of freedom and independence; in contrast to their stay in hospital, clients provided positive examples from their current housing situation (e.g., to pursue employment and education) - Residents spoke positively of the in-house supports they received, and that supports were better matched to their needs and in helping their recovery; residents and staff expressed an appreciation for support with medication management and transportation - Some residents recognized the benefits of being social and in living in a group setting, and others emphasized the importance of privacy and preferred to live alone; challenges around rules and restrictions, with the location of housing, and in experiencing discrimination from neighbours in the community were voiced |
| Centre for Addiction and Mental Health (2022)^36^ | *Alternate Level of Care High Support Housing Initiative*:   - Initiative developed by CAMH and other partners in response to the ALC housing crisis; considered a small-scale, localized project - With funding from the provincial government, the initiative includes high support housing, “step-up” housing, specialized clinical teams to assist with transition, and a flex fund | Not described | Target program population:  ALC patients | - At the time of report, 375 units of high support housing were available in Toronto - A projection analysis found that 66-80 additional high support housing units will be needed to house alternative level of care patients of an Ontario mental health and addictions hospital over the next 5 years |
| City of Edmonton (n.d.)^117^ | - Permanent supportive housing is described as congregate living with onsite, staff supports; includes secure units for people with mental illness and/or addictions - Differing from supported housing, people living in supportive housing receive some 24/7 onsite supported - Community-based Residential Facilities (CRBFs), or half-way houses, are described; CRBFs offer an alternative to “conventional forms of incarceration,” and act as a bridge between institution and community - CRBFs are subsets provide a structured living environment with 24-hour supervision, programs, and interventions | - CRBFs aim to promote the successful reintegration of offenders, using a gradual and structured transition to community | Target program population:   - Population varies, but can include people with mental illness and/or addictions | - At the time of report, there were approximately 5,200 permanent supportive housing units in Edmonton, with an estimated need for another 2,100 units; the greatest need being residential and lodge units - Participants support the idea of Community-Based Residential Facilities, and suggest that the cost of repeated incarceration is significantly most than that of providing a half-way house model |
| Community Support and Research Unit (2012)^48^ | Custodial Housing:   - Refers to models in which “residents are cared for in a standardized and routine fashion,” and typically operated for-profit by private landlords - The structure of custodial housing varies; these are commonly large, institutionalized settings (i.e., boarding homes), typically with shared rooms and fixed access to laundry, meals, and housekeeping services - 24-hour supervision provided - Staff in custodial facilities criticized for often lacking the appropriate training to provide mental health support   Alternative high support housing models are described, including *Supportive Housing in Peel’s (SHIP) High Support Housing Project, Habitat Services, the Eglington Project*, and *Edmond Place*:   - Habitat Services established in response to growing concerns about living conditions in privately-run boarding homes; the Eglington Project and Edmond Place are pilot projects stemming from Habitat Services in partnerships with community organizations, hospitals, and private landlords - SHIP High Support Housing project delivered by CAMH, and in partnership with SHIP; was a product of “progressive attitudes about best practice housing and supports” - The Eglinton Project, the Edmond Place, and SHIP High Support Housing Project provide single-site housing with 24-hour onsite support; accommodations vary, and include bachelor apartments and self-contained apartments - The Eglington Project, the Edmond Place, and SHIP High Support Housing Project use best practices to support tenants and health outcomes; services can include meal planning and skills development (e.g., cooking, laundry, independent living) | Custodial Housing:   - Criticized for prioritizing maintenance rather than recovery in residents, and for sharing many of the same assumptions that underlie older institutional models - Support is oriented towards care and dependency, rather than improving functioning or independence   Habitat Services, The Eglington Project, Edmond Place, and SHIP High Support Housing Project:   - Habitat Services set to improve housing services and standards - The Eglinton Project and the Edmond Place adopt a best practice approach to providing support - SHIP High Support Housing Project focused on integrating persons into the community, using a best practice model of recovery-oriented housing | Target program population:   - People with serious mental illness and high support needs - The Eglinton Project and the SHIP High Support Housing Project also accept referrals of hospitalized ALC clients | - At the time of report, ~6,025 custodial housing beds were available to people with mental illness in Ontario--this consists of 1,450 Homes for Special Care beds, 852 beds funded by Habitat Services, and ~3,723 domiciliary hostel beds; in total, for every ten supportive housing units in Ontario (10,000), there are six custodial housing beds - Claimed custodial housing is no longer the preferred option of high support housing; reasons for eliminating custodial housing are outlined, and include: the substantial costs associated with custodial housing, compared to other high support housing approaches; the belief that people can and do recover from serious mental illness; recognizing that consumers of mental health services have preferences for and can direct their care/recovery |
| Dorvil et al. (2003)^60^ | - Foster homes are described; host residences are expected to offer social protection, material services, and recreational activities - Allowances are determined by resident characteristics and service intensity, regulated by health and social services laws - Social service staff monitor and provide assistance - Variations in gender, age, and length of stay among residents; residents often have extended stays, some exceeding 15-years | - Original aim of foster homes was to provide patients with a normal environment conductive to functional integration into society - Criticisms have been noted, including homes resembling mini-asylums | Target program population:   - People with mental illness   Study sample (N=15):   - Gender: M=9, F=6 - Age range: 20-29 (n=1), 30-39 (n=3), 40-49 (n=7), 50-59 (n=3), 60-69 (n=1) | - Foster homes were, at times, perceived as substitute families, with managers fulfilling parental roles - Overreliance on professional support limits residents’ ability to act and integrate into the community network - Residents reported improved mental health, but remain dependent on services - Limited agency in decision-making, with choices often dictated by professionals; residents’ preferences for autonomous living are mixed, with a reluctance to leave the security of the foster home |
| Government of Ontario (2017)^128^ | High support housing initiatives are described, and include:  *District of Sault Ste. Marie Social Services Administration Board*:   - In partnership with the Sault. Ste. Marie Canadian Mental Health Association, has converted 10 social housing units to provide housing and 24/7 supports for clients with severe mental illness and/or addiction   *Supportive Housing in Peel (SHIP) High Support Housing Program* for Centre for Addiction and Mental Health alternate level of care patients:   - Self-contained, one-bedroom apartments - Multi-disciplinary teams provide flexible, customized, and personalized support - 24-hour support and recovery-focused services; services are more intensive initially, supporting transition into the community | District of Sault Ste. Marie Social Services Administration Board:   - Not described   Supportive Housing in Peel (SHIP) High Support Housing Program for Centre for Addiction and Mental Health alternate level of care patients:   - Guided by a rights-based, client-centered approach, emphasizing client choice in housing and supports | Target program population:   - People with severe mental illness and/or addictions; Complex health issues | - Best practices in supportive housing and related services/systems are described, and includes recommendations that supports be available 24/7, according to the range of individual needs (including onsite support) |
| Grant & Westheus (2008)^110^ | - Single-site high-support model is described, consisting of 30 bachelor apartments, offices, common lounge, eating and outdoor areas - Full-time caretaker lives onsite; program manager has an office onsite - Individual and group support, planned activities, and crisis intervention provided by resource workers; supportive counselling, crisis intervention, and recreational support provided by a peer mentor - Regularly planned activities, skills groups, and a meal program are offered to residents - Monthly meeting arranged for residents of the building; residents are expected to take an active role in meetings and the internal community | Models are “focused on building community” | Individuals with serious mental illness/mental health consumer-survivors  Characteristics only provided for sample of residents across housing models (N=27):   - Age range: Most participants being between 40 and 49 years of age - Gender: M=12, F=15 - Behavioural health: Schizophrenia being the diagnosis held by most tenants | - Residents of high support housing experienced significant improvements in the following areas: satisfaction with social support, perception of physical and mental health, and mastery over time, compared to residents of the low-support site - Residents of high support housing were satisfied with their living situation, and described several positive aspects of the housing: independence and choice; feeling connected and settled; safe, clean, comfortable living environment; a supportive, well-resourced program - Residents highlighted the meal plan as a negative of the high support model - Some differences in reported improvements by ethno-racial background |
| High Support Housing Consortium (2009)^114^ | - High Support Housing includes trained, onsite staff available 22-24 hours/day - Stay ranged from short- to long-term - Staff include: residential support workers, counsellors, personal support workers, nutritionists, program coordinators - Onsite services include: recreational activities, personal support, employment, counselling, crisis intervention, and life skills coaching and medication supports | Services described as recovery-oriented, focusing on client growth, holistic health, and emphasizing recovery as an individual process | Target program population:   - Persons with mental illness; often requires Axis I diagnosis, referrals from shelters or current homelessness, and motivation towards recovery | - Average per diem cost comparison: high support housing: $102.70 vs. acute inpatient bed: $1048.00 vs. psychiatric inpatient bed: $665.00 - 67% of high support housing providers reported insufficient resources, especially in staffing complement and skill; limited resources (e.g., financial) and access to community-based services reported - Shortage of high support housing stock and long waitlists noted; a pressing need for a wider range of high support housing, improving property management, and increased, full-time onsite staff emphasized |
| Homeward Trust Edmonton (2017)^116^ | *Ambrose Place (Nigerian Housing Ventures):*   - Funded by Homeward Trust, is an example of a permanent supportive housing considered “high intensity” - Independent units (private, or roommate accommodation) with common spaces (e.g., kitchens, community spaces) - 24/7 tenancy management; onsite availability of clinical staff - Residents supported through individual case management and goal setting; meals and housekeeping offered - Tenancy is not contingent on resident participation in programming and services | Homeward Trust applies a recovery-orientation to case planning and goal setting, and promotes transition to independent living | Target program population:   - Individuals with concurrent illnesses or addictions, who may require assistance with daily activities or who require end-of-life care | - A high demand for supportive housing units was identified in 2017 data, with 240 “high intensity” units required over the next 6 years - A previous, preliminary evaluation of Ambrose Place found reductions in the number of inpatient admissions, emergency department visits, and need for addiction- and mental health-related emergency medical services |
| Lesage et al. (2006)^99^ | *Seven Oaks*:   - Capacity of 38 beds - Emphasized individualized care planning based on mutually agreed-upon goals, outcome indicators, modelling, and support/education - Residents participate in daily planning, decision-making, meal preparation, and social programming   *South Hills:*   - Capacity of 40 beds in two buildings - Individuals live in groups of 5 in an apartment; each building is called a “house” - Apartments are equipped with a full kitchen, bathrooms, shared laundry, and individual rooms; meals provided - 24/7 staff, including various health professionals (nurses, occupational therapists, physicians, pharmacists, psychiatrists) - Staff act as facilitators, engaging in activities with patients and teaching them skills as needed - Residents engage in various activities, including volunteer work and events | Seven Oaks:   - Mission is to provide high-level care, treatment, and rehabilitation in a flexible, deinstitutionalized, and interactive community - Goals include: offering a safe environment, fostering personal responsibility; maintaining connections with the community, including family, friends, and local support services; involving residents, family, friends, and local support services as community resources   South Hills:   - Mission is to support individuals with serious and persistent mental illness in acquiring skills to live within their home communities; based on psychosocial rehabilitation principles | Target program population:   - Individuals with serious, complex, and/or rare mental disorders, requiring tertiary care; facility needs cannot be met by primary or secondary levels of care in the province (Seven Oaks) - Individuals with serious and persistent mental illness (South Hills) | - 51 patients were transferred to Seven Oaks from Riverview Hospital between 2001 and October 2003; 56 patients were admitted to South Hills from Riverview Hospital between April 2003 and May 2004 - Positive outcomes were identified among programs, including: successful transfer of all intended patients from Riverview Hospital; independent daily living skills development; satisfaction among residents in mental health services, housing, and personal safety; mostly positive clinical outcomes |
| Ministry of Mental Health and Addictions (2022)^127^ | The Complex Care Housing model delineates three tiers of housing and health support:  Intensive Supportive Housing:   - Includes Group home-type housing on Congregate Sites; offers intensified, coordinated services prioritizing individual autonomy - Services are flexible, not time-limited, and aim to match individuals with their preferred living environments in the community whenever possible   Transitional and Stabilization/Respite Services:   - Services are time-limited and shorter duration - Examples include transitional or “step down” services, providing care and housing during transition (e.g., bridging people discharged from acute care or forensic services)   Highest Intensity Housing:   - Offers 24/7 intensive oversight in a “home-like” setting for a small group - Prioritizes low client-to-staff ratios, specialized services, and preventing escalating needs - Transitions are supported based on client goals and participation is voluntary; focuses on community connection | - Aims to provide a “home-like” environment and avoid perpetuating institutionalization - While presented as a stepped model, acknowledges that individuals’ needs fluctuate over time and stives to mitigate the impact of these fluctuations on housing stability | Target program population:   - Adults aged 19 and above, who face substantial mental health, substance use, and other health challenges that are inadequately addressed by existing housing approaches | No outcomes were examined that are relevant to high support housing |
| Molina et al. (2018)^105^ | - Transitional Rehabilitation Housing (TRHP 1) provides one-to-one group support for 12 forensic patients living in a CMHA-operated building with 24/7, onsite staff - Staffing is provided by case managers during the day on weekdays, and residential support workers overnight and weekends - Access to psychiatry and nursing services offered by CAMH Forensic Outpatient Service - Length of stay varies, aims for 12 and 18 months - Other services include: social recreational and psycho-educational programs; symptom management; relapse prevention; medication support | Aid forensic patients in safely rejoining the community and guiding them towards community health and support services | Target program population:   - Individuals who have serious mental health issues, have been found Not Criminally Responsible, and are under the supervision of the Ontario Review Board | - Qualitative interviews with service users, staff members, and referral sources revealed the following strengths: TRHP 1 addresses the housing, mental health, and resource needs of service users; program aids in rehabilitation, community integration, and transition to mainstream services; program promotes well-being and skills development - Challenges include: limited access to health and counselling services; lack of physical activities and healthy food options - Various recommendations are provided, and include: upholding recovery-oriented care, focusing on service users’ needs, values, and choices; ensuring service providers are trained to effectively assess and respond to accessibility needs |
| Morrow et al. (2006)^126^ | Different housing options are described, and includes:  Residential/Family Care:   - Licensed care facilities providing 24-hour care and professional staff supervision, including supervised medication intake - Typically includes 6 to 10 beds - Category also includes family care beds, where a person lives with a family in the community   Enhanced Supported Independent Living (SIL):   - Non-profit; offered only by *the Portland Hotel Society* and *the Kettle Friendship Society*, includes one meal a day and 24-hour staffing within facility - Residents live in self-contained suites governed by the Residential Tenancy Act - Residents have access to intensive support services | Residential/Family Care:   - Not described   Enhanced (SIL):   - Combines autonomy for the client with access to community supports and protection against victimization | Target program population:   - Applicants must have a serious mental illness, be connected with a mental health team or psychiatrist, and comply with a medication regime | No outcomes were examined that are relevant to high support housing |
| Novac & Quance (1998)^56^ | *Habitat Services*:   - Regulated, private “board and care” home (i.e., “supportive, linked, high level of support”); provides permanent housing for individuals with psychiatric disabilities - Number of residents in a house varies from 9 to 35, with most of the residents sharing a bedroom; offers about 700 beds in total, with and expansion underway - Housing facilities are situated in traditional residential neighbourhoods, offering a “homey” atmosphere - Staff are available onsite, 24-hours a day to provide meals, clean the house, encourage personal care, and ensure basic security in buildings - Staff have basic training in relevant skills, and are not expected to provide crisis intervention   Additional examples of Shared Housing Units, with continuous support for populations of interest, are listed on pg. 25-26 | Not described | Target program population:   - People with psychiatric disabilities | - At time of report, there were 5,925 supportive housing units in Toronto (excluding frail elderly, people with developmental disabilities); about two-thirds of residents are psychiatrically disabled, homeless, or hard-to-house - Access to supportive housing is uneven, reflecting an absence of a co-ordinated infrastructure; assessment outlined a serious gap between the need and availability of supportive housing units in Toronto |
| Palermo et al. (2006)^130^ | Moderate Support Services:   - Staffing available 24/7 - Services include: accommodation, meals, case management, access to day programs and activities, medication monitoring   Intense Support Services:   - Staffing available 24/7 - 3 residents per home (facility I), and 4-10 residents per home (facility J) - Services include: meals, case management, counselling, access to day programs and activities; varies with level of care | To assist residents in daily tsks they may struggle with independently | Target program population:   - Adults with mental illness | Costs identified were sourced directly from housing providers:  Moderate Support Services –   - Cost per person/day: $43.33 - Funding Sources: Province (99%), Fundraising (1%)   Intense Support Services   - Cost per person/day: $150-350 (facility I); $100-200 (facility J) - Funding Sources: Province (99%), Fund-raising (1%) |
| Patterson et al. (2008)^76^ | Continuum of housing programs in British Columbia are described, and include:  Supported Hotels:   - Single Room Occupancy (SRO) hotels, leased or owned and managed by non-profit agencies - Residents pay reduced rent based on income, or non-profit society rents building - Onsite support and supervision up to 24-hours per day, provided by non-profit agency - Residents usually stay long-term (2+ years)   Supportive Housing - High-Level Support:   - Minimum two staff on duty 24/7; staff have some training in social work or psychiatric rehabilitation - Typically take the form of co-operatives and group homes - On-site medication management and on-site needle exchange is recommended - Psychiatric, medical and case management services must be hard-targeted to the building via an assertive outreach model | Supported Hotels:   - Not described   Supportive Housing – High-level Support:   - Often managed by non-profit agencies within a rehabilitation framework | Target program population:  Supported Hotels:   - Not described   Supportive Housing – High-level Support:   - Persons with complex health issues, including: severe and ongoing addictions, untreated or inadequately managed mental illness | No outcomes were examined that are relevant to high support housing |
| Sanford et al. (2022)^44^ | - Two models of supportive housing are identified: dedicated buildings with shared, or self-contained units - Supports are usually attached to the housing site - Building rules/eviction decisions are under control of the housing provider | Not described | Target program population:   - People with a range of experiences and needs, including mental health and substance use issues, histories of chronic homelessness, or extended stays in institutional settings | - Service users and subject matter experts indicated the need for greater supportive housing in Toronto, including expanding and investing in models with 24-hour onsite supports - At the end of fiscal year 2019, there were 960 individuals waiting for 24-hour support - Between 82-101 24-hour units will be needed over the next 10 years to accommodate ALC patients waiting for supportive housing - Prioritizing access to those requesting 24-hour or daily supports (i.e., applicants with highest needs), 6,400 units would be needed over 10 years (based on the City of Toronto’s target of creating 18,000 new supportive housing units over 10 years) |
| Serge et al. (2006)^90^ | Various housing programs are described, including:  *5616 Fraser Street Supported Housing Program, Vancouver:*   - 30 studio apartments; 12-18 months stays, extendable for up to 2 years - Staffed 24/7 with healthcare workers - Focused on rehabilitation, mental health support, improving life skills, and a encouraging drug/alcohol-free lifestyle   *Mainstay Residence, Winnipeg:*   - 34-bed facility with varying length of stay - Provides room, board, laundry facilities, and basic toiletries - Staffed 24/7, with a focus on supervision, medication management, and daily living assistance - Offers case management for transitioning out of the residence - Strict prohibition of drugs and alcohol   *Housing with Outreach, Mobile and Engagement Services (HOMES), Hamilton:*   - 181 units; buildings offer 12 or 24-hour onsite support - Provides varying levels of support including intensive case management, recovery support, personal care assistance, counseling, medication management, meal programs, and vocational support - Access to mental health professionals, pastoral support, and a trusteeship program | 5616 Fraser Street Supported Housing Program, Vancouver:   - Treatment focuses on abstinence and recovery - Emphasis on community integration and meaningful activities   Mainstay Residence, Winnipeg:   - Goal to provide a safe place for stabilization and transition to independent housing   HOMES, Hamilton:   - Focused on harm reduction and minimizing risks to physical health and safety - Embraces psychosocial rehabilitation and recovery-based approach | Target program population:  5616 Fraser Street Supported Housing Program, Vancouver:   - Persons with concurrent disorders who are homeless or at risk - Residents expected to be psychiatrically stable and receiving services from a mental health team - Requires applicants to be in recovery (alcohol and drugs-free) for at least 60 days   Mainstay Residence, Winnipeg:   - Men and women with substance use, mental health issues, co-occurring disorders, or at risk of homelessness   HOMES, Hamilton:   - Individuals with mental health illness who are homeless or at risk of homelessness | Report includes several case studies on high support housing, with results such as:   - Residents experiencing reduced substance use, with some achieving abstinence (*Mainstay*) - Residents demonstrated improvements in mental health, medication compliance, reduced hospitalizations, and enhanced physical health due to better nutrition and access to medical care (*Mainstay*) - Reduced hospitalizations among residents; improved ability among residents to manage daily activities and healthcare needs (*HOMES*) - Increased participation in social and recreational activities; vocational support resulted in employment opportunities (*Mainstay*, *HOMES*) - Satisfaction regarding staff support, housing quality, and social activities, across agencies |
| SHIP (2014)^129^ | - Initiative created to provide safe, stable housing options to meet the diverse and complex needs of people with mental illness - Multi-disciplinary, multi-service onsite staff provide immediate, responsive support - Tenants engage in monthly meetings, empowering individuals to reflect on their “value as tenants in the building and stakeholders in the community” - Promotes the enhanced quality of life and independent living | To reintegrate individuals requiring high levels of support into the community | Target program population:   - Individuals with mental illness, who may require high levels of support | No outcomes were examined that are relevant to high support housing |
| Sirotich et al. (2018)^118^ | Housing with 24-hour support is included in the report, but not described | Not described | Not described | - Among 12,733 applicants of supportive housing between January 2009-2015, 7% requested 24-hour support - People placed in units with 24-hour support tended to be: in the oldest and youngest age groups (under 25, over 55), have more inpatient hospital use, and be homeless while residing in hospital; applicants were more likely to require provision of meals, support with shopping and in managing specific symptoms - Based on the support needs associated with actual placement into units with 24-hour support, it was projected that applicants waiting for housing required approximately 300 units with 24-hour support |
| Suttor (2016)^57^ | - Congregate housing is diverse, and includes: high-support congregate housing (group homes), boarding homes, and shared houses or apartments operated by non-profit housing providers   Main program categories include:  CHPI Housing with Related Support (Domiciliary Hostel):   - Private-sector boarding homes, where service managers are required to set standards of care consistent with provincial criteria   *Homes for Special Care*:   - Private-sector boarding homes, with residents living in single or shared accommodations - Meals and housekeeping services, personal monetary allowances, and other types of supports are provided - Homes are regulated, with provisions set on licenses, inspections, standards, funding, and program administration   *Habitat Boarding Homes*:   - Subsidized, private boarding homes specific to the city of Toronto, Ontario - Program administration is carried out by Habitat Services under a legal agreement with the City - Operators are required to meet standards of housing and care as a condition of funding, and must provide access by support workers from a community support agency   Congregate High-Support Housing:   - Typically structured in congregate housing or clustered apartments - Commonly referred to as “group homes” and “residential care facilities,” includes beds (individual or shared rooms) in houses involving high staff-to-client ratios and supervised living | Boarding homes:   - Established as a way of regulating and improving room-and-board accommodation - Critiqued as custodial housing, failing to foster recovery, autonomy, and opportunity for residents   Congregate high-support housing:   - Part of a “linear residential continuum” approach to housing with supports; includes various levels of housing types and support services depending on resident needs | Target program population:   - People experiencing mental illness, addiction, or chronic homelessness, though target populations can vary by program categories | - In a 1999-2016 inventory of approximately 23,000 supportive housing units in Ontario, there were 5,300 boarding homes and an estimated 1,200 high support congregate housing units for people with mental illness or addictions |
| Trainor (1996)^58^ | - *Homes for Special Care* offers government-funded, privately-run accommodations - 160 licensed housing across the province; 88 homes have a capacity of 10 or fewer beds, often with a more “home-like” atmosphere and owners living onsite - Larger homes are typically run as businesses with managers and staff, while owners live elsewhere - Operators provide basic support for residents, including: assisting with medications; organizing recreational and social activities; handling laundry, housekeeping, and meals - “Fieldworkers” handle referrals, ensure that resident needs are met, manage home adjustments and issues, and act as a liaison with families and agencies | - Initially based on the assumption that individuals with serious mental illness would not improve, and would only deteriorate over time | Target program population:   - Patients discharged from psychiatric hospitals, who lacked immediate care and lodging options | Summary of Analysis:   - “Putting People First” strategy in Ontario targets hospital bed reductions, emphasizing the importance of good community housing, with Homes for Special Care being a crucial resource - The Homes for Special Care program was established when custodial care was perceived as the only option, hindering adaptation to new mental health approaches - Consumers preferred smaller, more independent settings; changes required in two area: broader mental health system support for Homes for Special Care residents, and within the program itself |
| Trainor et al. (2011)^31^ | Various program described, including:  *Stella Burry Community Services – Carew Lodge (St. John's, Newfoundland and Labrador):*   - Carew Lodge provides two transitional housing units for individuals re-entering the community under supervised parole - Transitional units offer enhanced supervision, security, and 24-hour staff support - On-site services include assistance with education, employment needs, advocacy support for income, provided by a community development worker   *Phoenix Residential Society (Regina, Saskatchewan)*   - Evolution from group homes to independent apartment living models - Cluster model with 16-unit apartment building; onsite staffing (24-hour) and case management - Partnership with health authority, CMHA, and Abilities Council for employment-related supports - Multidisciplinary team approach, involving mental health professionals, case managers, and peer support workers - Provides tailored supports, including mental health care, addiction services, and assistance with daily tasks   *Hamilton House: Post-Discharge Transition Program - CMHA (Calgary, Alberta)*   - Partnership between CMHA and Alberta Health Services; 8-bed facility for individuals at risk for prolonged hospitalization - 24-hour intensive support; individualized service plans, in-house groups for wellness and recovery, and collaboration with community resources - Staff includes mental health professionals, case managers, and peer support workers - Partnership benefits including on-site nurse and psychiatrist for community stabilization | Stella Burry Community Services – Carew Lodge (St. John's, Newfoundland and Labrador):   - Adopts a Housing First approach - Provides affordable housing to low-income individuals, including those with complex mental health needs, without requiring them to first address these issues   Phoenix Residential Society (Regina, Saskatchewan):   - Offers a range of housing models and support services tailored to individuals, emphasizing recovery-oriented care   Hamilton House: Post-Discharge Transition Program - CMHA(Calgary, Alberta):   - Offers an alternate level of support for individuals at risk for repeated or prolonged hospitalization, focusing on facilitating transition to long-term living arrangements within six months | Target program population:  Stella Burry Community Services – Carew Lodge (St. John's, Newfoundland and Labrador):   - Low-income individuals in St. John’s, including those with complex mental health needs   Phoenix Residential Society (Regina, Saskatchewan):   - People with psychiatric disorders, concurrent disorders (dual diagnoses), and acquired brain injury   Hamilton House: Post-Discharge Transition Program - CMHA(Calgary, Alberta):   - Individuals with severe mental illness at risk for repeated or prolonged hospitalization, who have been unsuccessful in attempts to live independently or access other housing supports | - The cost of housing and support models can vary considerably, but supportive housing with high levels of support is estimated to range from $82 to $115 per day - High support housing, particularly with round-the-clock on-site assistance, is identified as the most urgent unmet need; calls for additional units, especially for ALC clients |
| Wellesley Institute et al. (2020)^106^ | Mental Health and Justice Housing programs are described, and include:   - Transitional Rehabilitation Housing Program (TRHP) is dedicated supportive housing, offering 24-hour high support (daily structure, meal supervision) and case management as needed - Provides stability for clients after long institutionalization; clinicians assess skills in activities of daily living and risk, as well as monitor medication and form service plans | To transition patients with low- to moderate-risk from hospital to community settings | Target program population:   - Forensic mental health patients, who have been found Not Criminally Responsible by criminal courts, and are under purview of the Ontario Review Board | - Over 10% of ~5,000 mental health supporting housing units in Toronto, Ontario were in the Mental Health and Justice (MHJ) program targeting people with criminal justice involvement; 9% of existing MHJ program units provide 24-hour supports - Focus groups with residents and service providers identified strengths of MHJ housing, including providing immediate access to permanent housing with supports; 24/7 supports, access to inter-disciplinary care, meal programs, and supports with daily living were also identified - Recommended that justice-specific mental health and addictions supportive housing programs have multi-disciplinary teams and 24/7 capacity to meet complex support needs; dedicated (project-based, single-site) supportive housing can be advantageous for people with higher needs |
